# Measuring and assessing corruption in public health systems in low- and middle-income countries: a scoping review of methods

**DOI:** 10.1093/heapol/czaf113

**Published:** 2026-02-03

**Authors:** Bronté Anderson, Martin McKee, Prince Agwu, Dina Balabanova

**Affiliations:** Department of Global Health & Development, London School of Hygiene and Tropical Medicine alumni, 15-17 Tavistock Pl, London, WC1H 9SH, United Kingdom; Department of Health Services Research and Policy, London School of Hygiene and Tropical Medicine, 15-17 Tavistock Pl, London WC1H 9SH, United Kingdom; Education and Society, University of Dundee, Dundee, DD1 4HN, United Kingdom; Health Policy Research Group, University of Nigeria, 41001, Enugu, Nigeria; Department of Global Health & Development, London School of Hygiene and Tropical Medicine, 15-17 Tavistock Pl, London WC1H 9SH, United Kingdom

**Keywords:** corruption, anti-corruption, health systems, low- and middle-income countries, methods

## Abstract

Corruption in health systems has serious implications for health outcomes and equitable care. Although various methods exist to measure it, their application, purpose, effectiveness, and context have not yet been systematically consolidated to enable learning. We conducted a scoping review to identify empirical approaches used to measure health-sector corruption globally, with a focus on low- and middle-income countries. We examined the opportunities and challenges of these methods and developed a typology to guide future research. We searched Econlit, Embase, Global Health, Medline, Social Policy and Practice, Web of Science, and websites of international organisations focused on corruption research. Reference lists of included studies were also hand-searched. Two rounds of searches were conducted: first for studies published between 2000 and 2022, then for earlier publications dating back to 1993. Thirty-seven studies were narratively synthesised. Common methods included surveys, interviews, focus groups, and audits. Surveys were more common before 2000. Ethnography, investigative journalism, co-production, and crowdsourcing—though previously recommended—were rarely used or reported in the literature. Often, measuring corruption was not the primary aim, and methods were poorly described. Many lacked a clear definition of corruption or a theoretical grounding. Our review and typology highlight trade-offs between rigour, feasibility, and utility. As demand for evidence in this field grows, consensus on corruption definitions and sub-types is needed to guide study design and improve comparability across contexts. Promising directions include theory-informed mixed methods, context-sensitive designs, qualitative pilots, and interdisciplinary approaches.

Key messages

- There is increasing interest to measure health sector corruption—a complex and harmful set of practices—but the experience of using the methods is rarely considered.
- Our scoping review showed that health sector corruption in low- and middle-income countries is assessed mainly using surveys, interviews, focus groups, and audits and compliance reviews. Ethnography, investigative journalism, and crowdsourcing, while recommended previously, are less commonly used.
- Methods rarely arose from explicit theories and frameworks and are often not fully described.
- To strengthen research on corruption, a consensus on the definition of corruption practices, useful frameworks to guide study design, and employment of a broader range of methods, including from disciplines outside health, is needed.

## Background

Corruption is increasingly recognized as an important obstacle to achieving equitable health systems (Mackey et al. 2018). Yet, for many reasons, it is rare for something to be done about it. There are many reasons: it often takes place out of public view; those with the power to tackle it are complicit; it is perceived as deeply ingrained and therefore difficult to address; and in some cases, it is seen as a necessary evil that allows a dysfunctional system to operate. These characteristics also make it difficult to study.

Yet, unless researchers can characterise it and the harms it causes, identify the factors that allow it to emerge and persist, and design and evaluate measures to tackle it, it will be difficult to develop and implement the policies needed to reduce it. Previous foundational work has laid important groundwork for measuring health-sector corruption, such as Di Tella and Savedoff’s work for the Inter-American Development Bank, which applied a range of empirical methodologies in Latin American countries, including surveys and audits (Di Tella and Savedoff 2001). Their work remains a key reference for understanding the challenges of quantifying corruption in different contexts. Similarly, Leonard’s research in African settings has provided critical insights into the behavioural dimensions of corruption involving healthcare providers, using what were then innovative approaches such as direct observation and experimental designs (Leonard 1991). These studies have informed subsequent efforts to develop robust tools for measuring corruption in health systems.

Their work, and that of others, highlight the need to use context-specific methodologies that draw on a variety of disciplines and methods, and to adapt in ways that take account of how this phenomenon is actively concealed and people are unwilling to discuss it, perhaps because of fear of self-incrimination or violence. This calls for innovative ways to overcome these challenges.

While there are several methodological guides to studying corruption (Interdisciplinary Corruption Research Network 2022, USAID 2022) and insights from work in other sectors (Torsello and Venard 2016, Richards 2017, Luna-Pla and Nicolás-Carlock 2020), there are very few empirical studies that use rigorous designs to study corruption in the health sector. This gap dates back to the 1980s (Alam 1989, Campos et al. 1999) and was reiterated in a 2013 World Bank report (Filmer et al. 2013) and a 2016 Cochrane review of interventions to reduce corruption. The latter also noted the paucity of empirical studies on health corruption (Gaitonde et al. 2016). Although attempts have been made to develop such guidance, it has not yet been widely applied (Persson et al. 2012, Taylor 2018).

To address this situation, we conducted a scoping review of methods used to measure and characterise corruption in health systems in both high-income and low- and middle-income countries (LMICs), asking what can be learnt from their experience. We asked: ‘What empirical methods have been used to measure and characterise corruption in health systems across different country contexts, particularly in low- and middle-income countries (LMICs)?’ To answer this, we set four objectives: (1) review and create a typology of study designs and methods used to study health sector corruption across different countries, but especially in LMICs; (2) describe how they have been used, with what types of corruption, and in what contexts; (3) critically evaluate their strengths and weaknesses; and (4) propose recommendations based on the findings.

## Methods

We generated search terms from an initial rapid review of existing literature on corruption within and beyond the health sector (Ugur 2014, Molina et al. 2016, Onwujekwe et al. 2019, Naher et al. 2020). Items were included if they met the following criteria: used an empirical approach; published between 1993 and 2022; available electronically, in English, and in full text. We chose this inclusive approach to capture all relevant studies without imposing arbitrary start dates, given the scarcity of pre-2000 literature. The databases searched were Econlit, EMBASE, Global Health, MEDLINE, Social Policy and Practice, and Web of Science, and the search strategy is reported in Appendices A and B. The searches were run initially in 2021 and updated once in 2022. Reference lists were hand-searched for material not captured via formal searches. We excluded studies reporting on pharmacovigilance topics. As has been noted elsewhere, terminology on substandard, counterfeit, and falsified medicines is complex and raises issues that often cannot be definitively classified as corruption (Attaran et al. 2012).

We used the Covidence software to manage the results (Covidence Systematic Review Software, Veritas Health Innovation). Titles and abstracts were filtered according to the inclusion and exclusion criteria, and the remaining items were assessed in full-text. Two of the authors reviewed articles, and the other authors were consulted to resolve differences. The following data items were extracted: setting; study design; study purpose; sampling method; method(s) used to measure health sector corruption; nature of the relevant findings; and stated methodological strengths and limitations, including how data were analysed. Given that we sought to describe the methods used to measure health-sector corruption, a quantitative synthesis was not applicable, so the findings were synthesised narratively.

Regardless of scoping reviews not requiring quality assessment of methods, we still considered undertaking a formal assessment of qualitative studies, looking at instruments such as that developed by Long and Godfrey (2004). However, we concluded that our own data extraction tool captured those elements relevant to our limited aim, and there was a risk that we might exclude relevant studies because of problems with other criteria that were not pertinent to our objectives. This further reinforced our decision to include only empirical studies, as studies grounded in empirical data would naturally align with a degree of rigour, indicating quality.

While our formal scoping review was limited to peer-reviewed empirical studies, we also referenced selected reports from international organisations (e.g. WHO, World Bank, Transparency International) and examples from investigative journalism to contextualise the challenges of studying corruption in health systems (World Health Organization 2019, World Bank 2023, The Bureau of Investigative Journalism 2025, Transparency International 2025). These sources were not included in the synthesis or typology but were used to illustrate gaps in the academic literature and highlight methods that have been proposed or used outside the scientific domain. Their inclusion is intended to enrich the discussion and underscore the need for broader methodological innovation in future research.

Finally, our approach focuses exclusively on what and how empirical methods have been used to measure corruption in health systems, irrespective of specific health policy constructs. Thus, we do not seek to understand its impact on access to care, essential medicines, or other UHC-related outcomes. These are the subject of our ongoing research that will be published elsewhere.

## Results

### Search results and selection of studies

**Figure 1:**
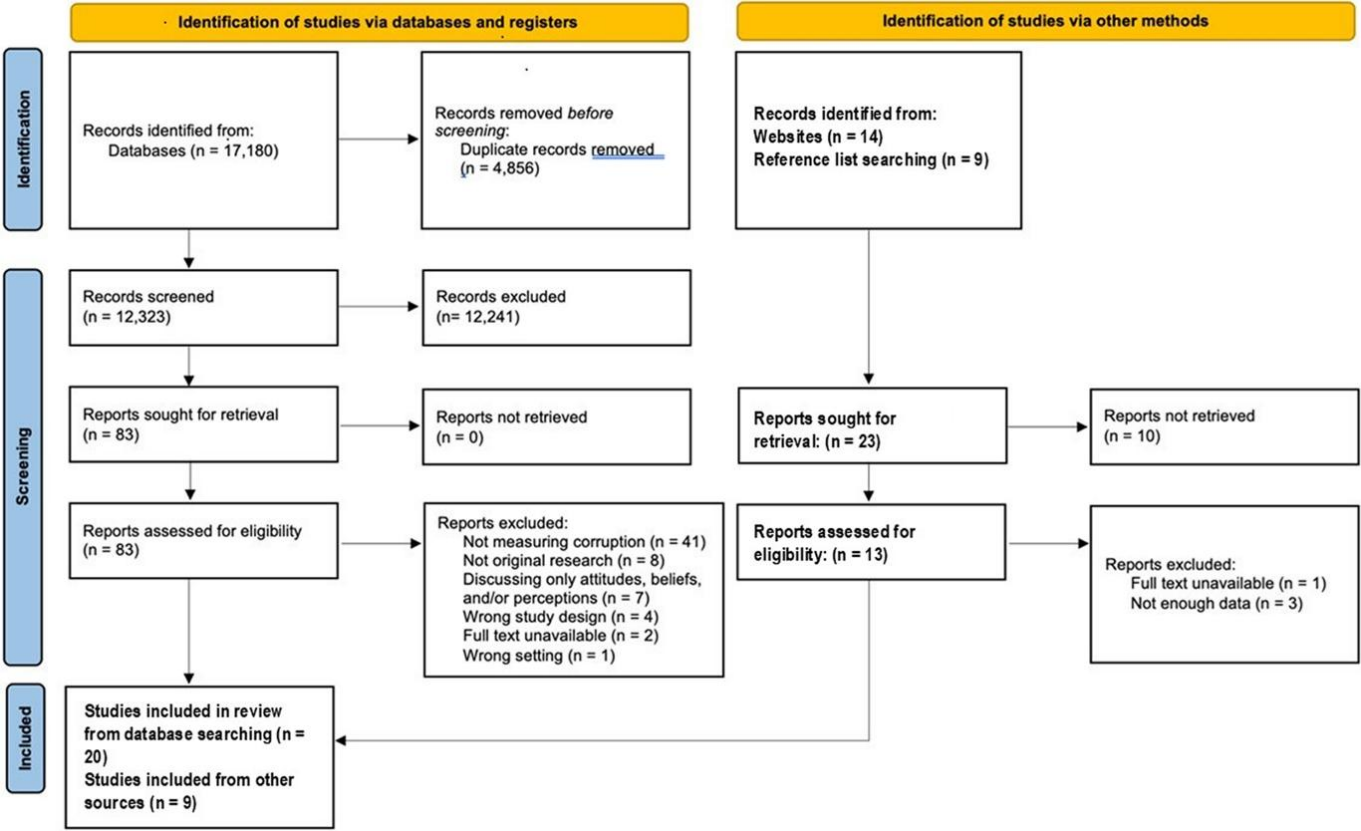
PRISMA flow chart.

We conducted our search in two parts. The initial search, which sought papers published after 2000, yielded 17 180 titles, reduced to 12 323 after duplicates were removed. Eighty-three were retained following title and abstract review, with 20 remaining after full-text review. Hand-searching identified an additional 19 articles, of which nine were included. By these means, 29 papers since 2000 were selected for inclusion in the synthesis. Figure 1 presents the PRISMA 2020 Flow Diagram for these (Page et al. 2021). Following reviewers’ request, we extended our search to the period before 2000. An initial electronic search suggested that such papers were very scarce (Alam 1989, Campos et al. 1999), and, given that we had seen the low sensitivity of this approach, with over one in five of the papers in the initial search being missed, we undertook a hand search of references in the later studies. Given that most authors of later studies would have used a range of methods to search for earlier studies, we considered this the most appropriate way to maximise sensitivity. Using this method, we retrieved an additional 11 studies published between 1993 and 1999. In total, our scoping review included 40 studies. It rapidly became clear that, while the year 2000 is an arbitrary date, there were apparent differences in papers published before and after it, so, where relevant, we have reported the results separately.

### Study characteristics

Of the 29 studies published after 2000, 21 (81%) were published since 2010 and were produced by 27 distinct author teams. The remaining 11 studies were published earlier, with nine between 1996 and 1999, and two between 1993 and 1995. Appendices C and D list the studies and their methods. Corruption was measured in many different ways, often as a secondary outcome of broader research. Commonly reported types of corrupt behaviour included absenteeism, fraud, drug theft and leakage, patient diversion, and informal payments or bribery. Some also mentioned substandard or counterfeit medicines, though these fell outside our scope. A few studies explored corrupt behaviours without specifying a type.

Most studies were conducted in Africa, Asia, and Eastern Europe, with a few from the Americas and one from Australia. Three pre-2000 studies analysed surveys on the impact of corruption on health. Post-2000 studies were mainly observational, using qualitative methods such as interviews, participant observation, and focus groups. Quantitative approaches included descriptive surveys, audits, compliance reviews, content analysis, and a discrete-choice experiment.

**Figure 2:**
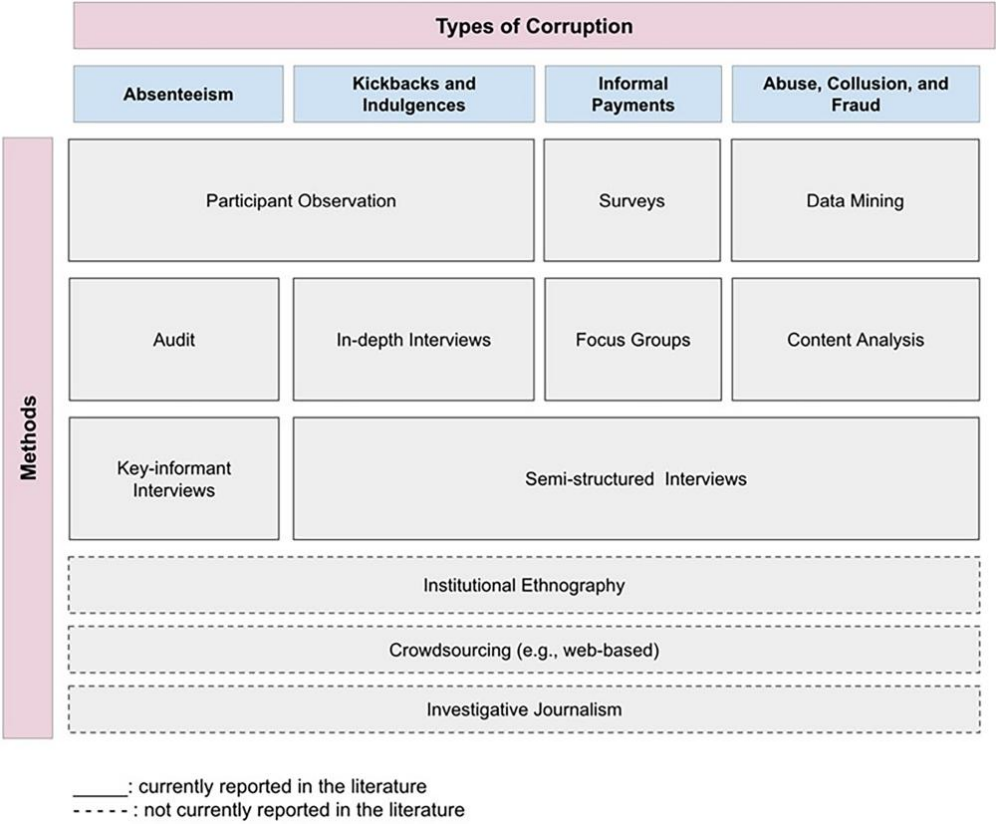
Methods for assessing different forms of corruption: current and potential.

We categorised methods by their characteristics (Fig. 2), including those used to measure different types of health-sector corruption and those proposed in principle but not yet applied empirically. These ‘missing’ methods are displayed under the types of health-sector corruption for which they may be most suitable.

### Theoretical and conceptual frameworks

Among the studies published since 2000, 13 were guided by theory or conceptual frameworks, at least to some extent, even if this was not explicitly stated. These comprised the economic model of human behaviours (Garcia-Prado and Chawla 2006, Lindkvist 2013), random utility theory (Angell et al. 2021) a modified version of Kumaranayake’s conceptual model of corruption (depicting regulatory frameworks, specific instruments, and efforts of understanding dynamics of practice, as they pertain to regulating healthcare providers) (Hongoro and Kumaranayake 2000), developmental governance and political economy (Hutchinson et al. 2020, Angell et al. 2021), the two-level principal-agent client model(Huss et al. 2011), Bourdieu’s theory of capital (Huss et al. 2011), agency theory (Rispel et al. 2016), the cognitive-behavioural model (Stepurko et al. 2015), grounded theory (Vian et al. 2013), the utility model of choice (Chaudhury and Hammer 2004), critical realism (considered an analytical approach to understanding reality rather than a theory) (Odii et al. 2022), and political settlement theory (Binyaruka et al. 2021). A theory of change was also used to contextualise and frame some results (Schaaf and Dasgupta 2019). These theories mostly view corruption as resulting from deprivation, system strain, and self-interested rational decision-making, and conclude that it is difficult to elucidate and operationalise.

None of the pre-2000 studies relied on mainstream theories. The closest was Asiimwe et al. (1997), who utilised a conceptual framework that was not rooted in theories or models. They employed a conceptual framework centred on informal health markets and financing policy. This framework mapped relationships between health worker survival strategies, payment irregularities, and service delivery outcomes. While not grounded in formal theory, it structured data collection and interpretation, highlighting how economic pressures shape informal practices that undermine health system integrity.

Several studies tested hypotheses linking corruption to health system performance and broader development outcomes. For example, Tanzi and Davoodi (1998) applied macroeconomic models to examine how corruption distorts public investment and reduces growth, while Mauro (1995) used econometric models to quantify the impact of corruption on GDP and sectoral allocations, including health. McPake et al. (1999) explored informal economic activities of health workers, hypothesizing that payment irregularities drive coping behaviours such as absenteeism and diversion of resources. These models provided early predictive and quantitative insights into systemic effects of corruption.

### Surveys

Of the 29 studies published from the year 2000, four involved analysis of surveys (Gopakumar and Balakrishnan 2000, Stepurko et al. 2015, Angell et al. 2021, Binyaruka et al. 2021). Stepurko et al. (2015) explored informal payments in a survey that used multi-stage random sampling, developed by a multi-country research team, pre-tested, and reviewed by external experts, which contained questions regarding past experience with and attitudes to informal payments. This team analysed another survey to investigate informal payments (Stepurko et al. 2017). Here, questions spanned characteristics and reasons for respondents’ recent doctor visits and hospitalisations. Both studies reported the prevalence and types of informal payments, perceptions of this practice, and changes over time. The cross-sectional design and short study periods reduced the risk of recall bias. Limitations included an inability to assess the magnitude of informal payments and the need for face-to-face survey administration. Though the surveys collected data on respondents’ past payments and most recent healthcare consumption, they only measured the magnitude of payments at a single point in time. Self-administration may have been preferable to face-to-face interviews, given the sensitive nature of informal payments, although most respondents answered all questions.

Angell et al. (2021), drawing on a random utility theory and employing a developmental governance approach, conducted a discrete choice experiment with 12 unlabelled choice sets. The attributes were generated from a literature review, qualitative interviews, and a consensus-building workshop with key stakeholders. They included community relationships, security, attendance-based policies, and incentive payments. While recognising the rigour of this theory-driven approach and its identification of doctors’ preferences regarding attendance policies and job regulations, discrete choice experiment results are based on hypothetical situations rather than experience, so some caution is needed. Binyaruka et al. (2021) surveyed informal payments in Tanzania, including a discrete-choice experiment. Its cross-sectional survey measured the proportions of healthcare workers who had accepted informal payments and the determinants of their practices. This provided insights into the scale and nature of informal payments and suggested potential policy remedies.

In the earlier period, Lennane (1993) examined whistleblowing in Australia, finding that corrupt officials often held enough power to block accountability. Whistleblowers were frequently dismissed as mentally unstable and referred for treatment. Many who had previously used whistleblowing services were reluctant to do so again, fearing victimisation and emotional distress. In Latvia, Anderson (1998) used household surveys to explore public understanding of corruption, its definitions, manifestations, and consequences. However, the author noted that these surveys primarily captured data on low-level corruption.

There are early reports of surveys being used to provide accountability from the Dominican Republic and Bolivia (Lewis et al. 1996, Gray-Molina et al. 1999), in ways that can challenge existing public data. For example, in the former country, health workers recorded doctors’ presence in hospitals, but surveys of service users found that official data overstated attendance.

Early research drew on large datasets compiled by organisations such as the World Bank and Business International, covering at least 90 countries (Mauro 1995, Mauro 1998, Tanzi and Davoodi 1998). Although these datasets were not well described in the published studies, the authors aimed to establish causal links between corruption and governance, including its impact on healthcare provision and outcomes. Their models laid the foundation for a significant strand of research into the complexities of health-sector corruption—its nature, types, drivers, effects, and potential responses. However, while these studies documented the prevalence and consequences of corruption, they offered few practical solutions.

### Audits and compliance reviews

Several studies analysed existing audit data (Garcia-Prado and Chawla 2006, Avelino et al. 2014, Joudaki et al. 2015 ). One used health insurance data to detect fraud, abuse, and collusion in physician claims in Iran (Joudaki et al. 2015). Indicators of doctor-related corruption were developed from outpatient insurance claims and validated through expert interviews and literature review. However, details on data collection were limited, and while some physicians were flagged for suspected fraud, the study noted key limitations. Existing information systems may lack comprehensive data, and critical elements related to fraud and abuse are often poorly recorded. Additionally, fragmented insurance markets pose challenges, and in low-resource settings, analysing physicians rather than claims may be more effective.

Avelino et al. (2014) assessed health-sector corruption in Brazil using audit data from over 900 municipalities. They used irregularities as indicators of potential corruption, providing insights into its prevalence and scale. Although they did not discuss methodological or practical limitations, they noted that audit reports can reduce the subjectivity often associated with corruption indicators, provided the reports are comprehensive and accurate.

Garcia-Prado and Chawla (2006) used hospital performance and staffing data to examine absenteeism. They measured cumulative sick-leave days to evaluate the impact of hospital management reforms. However, their approach could not distinguish between voluntary and involuntary absences, limiting its value.

One study, reported in two linked papers, used unannounced visits to rural healthcare centres to measure absenteeism (Chaudhury and Hammer 2004). Researchers recorded staff presence during morning and afternoon visits and asked present workers about their absent colleagues. Data were analysed by socio-demographic factors, residence, road access, and rural electrification. However, the findings were mainly descriptive, and causal relationships were not established. The infrequency of surprise visits limited insights into reasons for absenteeism. Additionally, the method required mobile research teams, posing challenges in areas with poor transport infrastructure.

### Interviews

None of the pre-2000 studies relied solely on qualitative interviews. Among those post-2000, nine included interviews. However, several did not report how they developed interview guides (Bouchard et al. 2012, Agwu et al. 2020, Ramesh et al. 2022). Authors noted how open-ended interviews may be less subject to bias because it is harder to impose preconceived theories and beliefs on the data. However, some did express concern about snowball sampling, which was widely used, may introduce bias since people with similar views may be more likely to know each other.

Two studies used interviews to assess awareness of healthcare laws and regulations related to corruption (Hongoro and Kumaranayake 2000, Huss et al. 2011). In Zimbabwe, Hongoro and Kumaranayake (2000) asked stakeholders open-ended questions about performance rules and regulations, revealing knowledge gaps, poor compliance, overlapping corrupt practices, and their underlying causes. Although the study included a diverse range of informants, from public and private sectors, local authorities, associations, and NGOs, the sampling method was not described, though it appeared purposive. Similarly, Huss et al. (2011) conducted semi-structured interviews with laypersons and healthcare staff to explore corrupt and illegal practices. Questions focused on health-sector functions, with prompts based on examples from key informants. The study uncovered numerous instances of poor governance involving actors such as politicians and service users. Limitations included potential bias in responses and difficulty distinguishing corruption from poor judgment.

Lindkvist also used interviews (2013) to examine informal payments. Patients were asked whether they agree with the following: ‘Usually, if a patient gives the doctor a gift, s/he will get better-quality services’. While this provided insight into the prevalence of informal payments, it had limitations, as patients may be reluctant to share information about them and may even be entirely unaware that they are informal.

Vian et al. (2013) also used semi-structured interviews, but to assess abuses of allowances. Participants (health care workers and ministry informants) chose the location of their interviews so that they could speak freely. The data were analysed thematically to extract information on the existence of corruption, its types, and its correlates. Yet informants sometimes discussed these practices in cultural terms, for example, ‘what people here do.’ Consequently, it may be challenging for researchers to collect data if they are detached from the participants’ setting(s) and culture(s). It was also noted that this method did not provide evidence on the scope of abuse and may be of limited use to inform interventions.

Although measuring health-sector corruption was not their primary aim, Yellappa et al. (2017) conducted in-depth interviews that yielded limited data on kickbacks to healthcare workers. As in Vian’s study, interviewees chose the location, typically their homes. Limitations included the framing of health-seeking behaviours from patient and private provider perspectives, potential recall bias in patient narratives, and the possibility that patients, contacted by programme staff, may have hesitated to disclose sensitive information.

Lastly, Hutchinson et al. (2020) carried out extensive in-depth interviews to explore absenteeism amongst Bangladeshi doctors in rural areas. They found absenteeism to be a common coping response to challenging circumstances, but they emphasised the need for nuance to capture the challenges specific to doctors with different social networks and access to resources, as there was significant variability in behaviours and possible solutions.

### Focus groups

As the only study of its kind, Lindelow and Serneels (2006) conducted semi-structured focus groups to explore factors affecting healthcare worker performance in Ethiopia. Multiple sessions were held with both healthcare workers and users, guided by a checklist of topics (not described). This approach generated insights into the prevalence, types, and drivers of corruption. Focus groups proved effective in eliciting views on sensitive issues that might not surface in individual interviews. However, ensuring representative participation is essential for the validity of findings.

### Mixed methods

Five studies combined methods (Balabanova and McKee 2002, Ackers et al. 2016, Rispel et al. 2016, Schaaf and Dasgupta 2019, Tumlinson et al. 2022). Common justifications included the desire to compare and contrast quantitative and qualitative findings and to glean data from multiple sources. Ackers et al. (2016) audited health performance data and conducted interviews and focus groups on absenteeism and moonlighting by Ugandan healthcare workers. Although their methods were not described in detail, they found that both were common and identified those actors involved and why. Likewise, Tumlinson et al. (2022) conducted random site visits and carried out focus groups to ascertain the prevalence of absenteeism in Kenyan family planning facilities. This established the prevalence of absent staff, their characteristics, and whether they were legitimate. One limitation was that the consent process may have increased attendance.

Rispel et al. (2016) triangulated data from audit reports, key-informant interviews, and print media content analysis to examine corruption in South Africa’s health sector. Audit reports used irregular expenditures as proxies for corruption and tracked trends over time. Interviews explored perceptions of corruption, both sector-wide and within individual work settings, and identified actors perceived as more vulnerable. The media analysis drew from a national news article registry. Together, these methods revealed the extent, locations, actors, and drivers of corruption. However, some irregular expenditures may have been legitimate, potentially inflating corruption estimates. Interview responses reflected individual interpretations of ‘corruption’ and were limited to a single point in time. Despite these limitations, findings across all three methods were consistent.

Schaaf and Dasgupta (2019) used multiple methods to investigate informal payments in India. Their approach included structured interviews with programme implementers and government officials at facility and district levels, focus group discussions with programme members and staff, participant observation in healthcare settings, and a review of programme documents. These methods revealed the prevalence of corruption, the conditions under which it occurs, and its underlying causes. Although methodological strengths and limitations were not discussed, the findings aligned with existing literature.

In Bulgaria, Balabanova and McKee (2002) combined surveys and focus groups to examine the scale and drivers of informal payments, using the OECD’s definition of the informal economy. Informal payments were defined as instances where staff solicited unnecessary payments or altered decisions to favour specific clients. Surveys drew on instruments from the World Bank Living Standards Measurement Study and the Health Survey for England, collecting data on demographics, health status, healthcare use, and spending. Semi-structured interviews and focus groups with healthcare staff and users complemented the survey by providing deeper insights into the meanings and contexts of informal payments. These methods identified their prevalence, locations, and correlates. The limitations were similar to those noted by Stepurko et al. (2017) and included potential underreporting due to the sensitivity of the topic, recall bias, and the emergence of new insights during the study that could have helped distinguish between payment types if known earlier.

Mixed-methods studies gained increasing popularity during the 1990s, coinciding with a growth in studies looking explicitly at corruption, eventually including issues specific to the health sector. These mixed-methods studies were often in response to earlier studies that argued that a single method was inadequate to capture the complex nature of corruption (Lennane 1993, Mauro 1995). Thus, we found four published studies from the late 1990s that used a mixed-method approach, encompassing survey analyses and a range of qualitative methods (Asiimwe et al. 1997, Ablo and Reinikka 1998, Kaufmann et al. 1998, McPake et al. 1999). These approaches produced more nuanced data and allowed researchers to modify questions in surveys, and even the data collection strategies (Asiimwe et al. 1997, Kaufmann et al. 1998). In Uganda, mixed methods won the trust of policymakers and were used by the government to design and evaluate interventions to track budget appropriations to the health sector and to tackle informal payments and diversion of health consumables (Ablo and Reinikka 1998).

### Assessing illegal market practices

Gong et al. (2020) used simulated clients to investigate illegal antibiotic sales at in-person and online pharmacies in China. Trained researchers posed as customers without prescriptions and attempted to purchase antibiotics, then completed surveys noting whether pharmacies displayed signs prohibiting illegal dispensing. While the study provided useful insights, it may have underestimated the true prevalence of illegal sales, as it focused only on antibiotics—a small subset of total medicine sales.

## Discussion

Although various methodologies have been proposed for studying corruption, our review shows that experience of applying them in research on the health sector is limited, but growing. Here, we critically review the methods used, their purposes, and their contexts. We found 40 articles and narratively synthesized them. Most were published after 2010, so although discourse on corruption is not new, efforts to document it empirically are relatively recent.

### Critique

A lack of clear conceptualisation limited many studies. While corruption was broadly understood as the misuse of funds or resources for personal gain, specific forms were often poorly defined and operationalised. With one exception, studies using surveys or qualitative methods relied on open-ended questions or multiple-choice formats without precise definitions. This lack of standardisation reflects both the field's evolving nature and the influence of context. As Vian (2008) noted, definitions of corruption vary across and within countries, shaped by local norms, attitudes, personalities, and ethical beliefs. For example, what is considered bribery in one setting may be viewed as reciprocity in another.

The conceptualisation of health-sector corruption is further complicated by its potential role as a coping mechanism, as documented by Marquette and Peiffer (2019). Several studies identified alternative explanations for behaviours that appeared corrupt but could not be clearly distinguished with the available data. These included unintentional absences, resource constraints, and economic hardship.

Despite the involvement of disciplines such as cognitive and behavioural sciences, behavioural economics, political science, and sociology, few studies were grounded in theoretical frameworks. This absence is notable, as Wise and Shaffer (2015) highlight the challenges of interpreting qualitative data without theory—particularly in distinguishing meaningful findings from less relevant ones. Theory is especially critical when analysing large datasets, as it provides a foundation for interpreting results and identifying actionable insights. A theory-informed approach to study design and methodology helps prevent misinterpretation and ensures important findings are not overlooked.

The limited use of ethnographic methods was unexpected, given their potential to deepen understanding of how corruption is conceptualised across contexts. Ethnography offers insights into the perceived morality of corruption, the role of informality—often a driver of corrupt practices—and provides a processual, interpretivist lens for inquiry (Torsello and Venard 2016). In health systems research, ethnography can help situate corruption within broader systemic and societal contexts, revealing upstream factors such as norms, attitudes, and beliefs beyond individual actors like patients or healthcare workers. This is essential for designing interventions that are both acceptable and accessible. This gap appears to have originated before 2000, when research largely focused on establishing causal links between corruption and development outcomes, such as public investment impacts on health (Mauro 1995). A more direct focus on health-sector corruption emerged in the late 1990s, particularly in Africa, South America, and the Caribbean (Lewis et al. 1996, Gray-Molina et al. 1999, McPake et al. 1999), but studies continued to rely on interviews and surveys, overlooking ethnographic approaches.

### Appraisal of the research methods used to study health sector corruption

**Figure 3:**
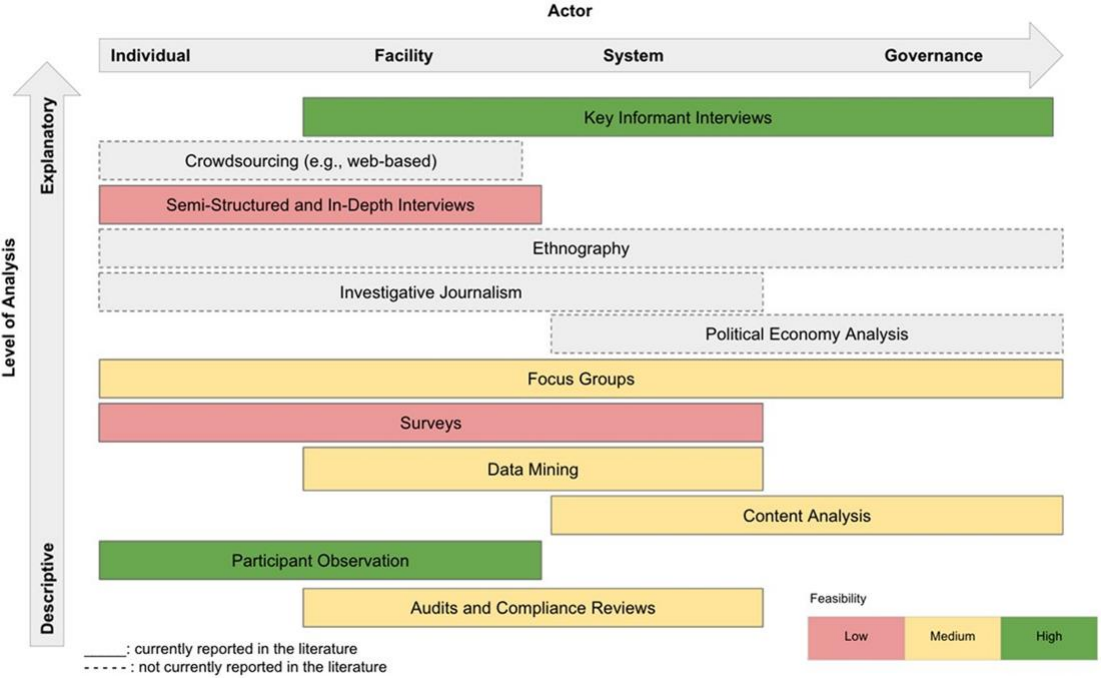
Typology of research methods which have been used to measure health sector corruption.

Figure 3 categorises the identified methods vertically by their analytical depth, from descriptive to explanatory, and horizontally by level of analysis, ranging from individuals to facilities, health systems, and broader governance. Each method is assessed for feasibility: low, medium, or high.

Semi-structured and in-depth interviews, along with surveys, were rated as low feasibility due to their high development costs. Despite this, they have been used across diverse settings, including rural and urban public and private healthcare facilities. These methods provide both descriptive insights into health-sector corruption and explanatory data on its scale, causes, and contributing factors. However, they are limited in quantifying corruption, have low generalizability, and face challenges in recruiting participants due to the sensitivity of the topic. As such, they may be most effective when combined with quantitative approaches. Surveys, in particular, are also resource-intensive.

More feasible methods included focus groups, data mining, content analysis, and audit and compliance reviews. These approaches, and particularly the latter three, have been used to describe forms of corruption such as fraud and abuse. While relatively inexpensive, they have methodological limitations. Focus groups are cost-effective but may struggle to uncover sensitive issues. In contrast, data mining, content analysis, and audits avoid the challenges of direct participant engagement, reducing risks of social desirability and recall bias. Leveraging existing data are also more affordable than collecting new data. However, these methods may miss high-level corruption involving data concealment or falsification, and poor data quality, especially in low-resource settings, can undermine validity.

Participant observation and key informant interviews were rated as highly feasible. These methods have been used to explore a wide range of corrupt practices. Participant observation allows researchers to understand corruption from the perspective of those affected and offers flexibility to explore emerging themes. It also enables unobtrusive observation, though it carries a risk of interpretive bias. Key informant interviews are similarly feasible, having been applied across individual and governance levels. They do not require validated tools or rely on existing data quality, making them adaptable and widely applicable.

Prior to 2000, the focus had been on establishing causal links between corruption and its effects on public investments. Using national surveys and interviews to investigate these issues, especially their impact on health, made sense (Lewis et al. 1996, Asiimwe et al. 1997, Ablo and Reinikka 1998, Kaufmann et al. 1998). Although most of these studies relied mainly on surveys, interviews, modelling, and some observation, there remained room for broader approaches. For instance, in Uganda, interventions were designed using findings from mixed-methods research but did not involve co-production, which would have allowed service users and frontline practitioners to contribute to their design effectively (Ablo and Reinikka 1998). This approach misses key elements of best practice. After 2000, we observed a decline in the use of quantitative models explicitly tailored to health sector corruption.

Ethnography, investigative journalism, and crowdsourcing of information—methods recommended for measuring corruption in various settings, including health facilities—were notably absent from the scientific literature (Vian 2008, Interdisciplinary Corruption Research Network 2022, USAID 2022). While investigative journalism is not typically published in academic outlets, its exclusion raises questions about the boundaries of academic research on corruption.

At the individual and facility levels, crowdsourcing may be particularly useful for capturing the prevalence, experiences, and drivers of corrupt behaviours, especially in contexts where surveys are impractical. Ethnography and political economy analysis offer upstream perspectives, exploring the anthropological and political dimensions that shape and sustain health-sector corruption. Though resource-intensive, these methods can yield rich, context-sensitive data to inform policy and intervention design.

Investigative journalism deserves special mention. In recent years, it has produced some of the most impactful revelations about corruption, notably through exposés such as the Panama Papers and Paradise Papers (Berglez and Gearing 2018, O’Donovan et al. 2019). Other valuable insights have come from public interest litigation, exemplified by the work of the Good Law Project in the United Kingdom, which has exposed widespread procurement abuses during the pandemic (McKee 2021, Good Law Project 2023). However, researchers often face barriers to using these methods, especially when ethics committees impose requirements that fail to appreciate the challenges of observing activities subjects seek to conceal. These methods can also be extremely dangerous, with several high-profile murders of journalists investigating corruption.

### Further recommendations for methodological improvements

Based on our critical analysis of the theoretical frameworks used in the reviewed studies, we offer several recommendations for future research. Studies drawing on agency theory, political economy, and developmental governance highlighted the systemic and relational nature of corruption, underscoring the need for context-sensitive, theory-driven approaches. The use of grounded theory and critical realism in some studies emphasized corruption as a socially constructed and deeply embedded phenomenon, supporting the case for ethnographic and interpretive methods.

Frameworks such as the principal-agent model and utility theory point to the importance of capturing individual decision-making and incentives, areas well-suited to mixed-method designs. Synthesizing these insights, we recommend that future research be explicitly guided by conceptual frameworks that reflect the complexity of corruption and its manifestations in health systems.

First, future studies should explore how definitions of health-sector corruption vary across groups and settings. While some research has examined beliefs and attitudes, little is known about how differing definitions influence the measurement, monitoring, and evaluation of corruption. Developing a consensus definition and relevant indicators will require coordinated, cross-sectoral efforts.

The methodological insights from this review support the use of theory-driven mixed-methods approaches. Beyond guiding inquiry and interpretation, mixed methods help reconcile contradictions between qualitative and quantitative findings and often produce richer, more actionable data. They may also help overcome logistical and resource constraints, particularly in low- and middle-income countries (LMICs).

Given the limited literature on health-sector corruption, researchers should consider how other disciplines—such as economics, big data, public administration, law, and psychology—have addressed similar methodological challenges. However, context must remain central, allowing for adaptation to local realities. For example, Ablo and Reinikka (1998) found corruption easier to study in the education sector than in health, due to the latter’s complexity and the number of actors involved. Cultural and behavioural differences also matter. Studies reported greater openness in discussing corruption in Africa compared to Australia and Europe (Lennane 1993, Asiimwe et al. 1997, Anderson 1998), highlighting the importance of tailoring research approaches to local norms and sensitivities.

### Study limitations

This review has several limitations. First, although we examined the extent to which theoretical or conceptual frameworks informed studies, we did not apply a formal guideline to analyse these frameworks. Instead, we identified and summarised those referenced, ranging from economic models and agency theory to political economy and grounded theory, highlighting their relevance to corruption research. Our approach was descriptive, aimed at showcasing the diversity and limitations in theoretical engagement. A future step could involve developing a structured framework for conceptual analysis, such as those used in realist reviews or theory-driven evaluations. However, this was beyond the scope of our review.

As a scoping review, our primary goal was to map the range of empirical methods used to study health-sector corruption, not to conduct a comprehensive quality appraisal of all study types. Given the prominence of observational studies, we applied a modified version of the QATSO. Researchers seeking deeper analysis could consider using tools designed for evaluating qualitative research.

## Conclusion

Given the growing demand for research evidence on health sector corruption, we critically reviewed the literature for studies reporting on the application of empirical methods for its measurement in LMICs. While a diversity of methods has been recommended (USAID 2022), few have been used in practice. Commonly used methods include surveys, interviews, focus groups, and audit and compliance reviews, seeking to measure specific types of corruption, as well as corruption more broadly. The typology presented in this review organises these research methods according to perceived feasibility. It recognises the need for trade-offs between feasibility, rigour, and usefulness, and presents pragmatic guidance for researchers designing studies of corruption and policy makers commissioning and using research. It is recommended that future studies adopt theory-informed mixed-methods and consider a broad range of methods for studying corruption across disciplines. Further investigation is warranted to reach consensus on the definition of corruption and its types, the indicators of corruption, and the utility of methods beyond health.

## Supplementary Material

czaf113_Supplementary_Data

## Data Availability

All data relevant to the study are included in the article.

## Adherence to reporting guidelines

We report our results according to recognised reporting guidelines for health research. Our Prisma chart is uploaded as a Supplementary File.

## Reflexivity statement

B.A. conducted the work and developed this paper drawing on her Masters summer project at LSHTM after discussing the initial idea with the other authors. She is a young female researcher with a strong interest in methods to assess corruption and understand how it impacts on systems performance and health outcomes and hopes to do further work on global health policy in the future. Developing the paper involved work beyond what was required for her summer project and represents an original contribution: D.B. and M.M. D.B. and M.M. supported her and provided mentoring on all aspects of the review. P.A. played a critical role in conducting a major revision, rethinking the content and interpretation.
