# Supplementary material for: Measuring and assessing corruption in public health systems in low- and middle-income countries: a scoping review of methods
Source: Health Policy Plan. 2026 Feb 3;41(3):460–70. doi: 10.1093/heapol/czaf113 (PMC12972675; doi:10.1093/heapol/czaf113)
Supplement: czaf113_Supplementary_Data [file czaf113_supplementary_data.docx]

**Appendix A. Search Concepts and Terms**

| **Search Concepts and Terms*** | | |
| --- | --- | --- |
| **Types of Corruption** | **Measurement Methods** | **Health Sector** |
| Corruption  Indulgences  Absenteeism  Bribery  Kickbacks  Collusion  Theft  Diversion  Pseudo-trials  Falsification  Counterfeit  Nepotism  Cronyism  Informal payments  Unnecessary referrals  Informal practice  Illicit practice  Dishonesty  Skimming  Speed-up money  Grease money  Rent-seeking  Embezzlement  Extortion  Misappropriation  Bid rigging  Price fixing  Favouritism  Abuse of power  Blackmail  Clientelism  Patronage  White collar crime | Methods  Measurement  Detection  Surveillance  Metrics  Indicators  Survey  Interview  Scale  Audit  Monitor  Inquiry  Focus group  Discussion group  Questionnaire  Experiment  Trial  Test  Observation  Assess  Score  Qualitative  Quantitative | Health sector  Health care  Health setting  Health facility  Health service  Health system  Health care personnel  Hospitals  Clinics  Healthcare management  Healthcare administration  Providers  Doctors  Nurses  Pharmacists  Medics |

*Searches were limited to LMICs through the London School of Hygiene and Tropical Medicine’s pre-set expert LMIC searches.

**Appendix B. Database Search Strategies**

Ovid MEDLINE(R) ALL <1946 to July 23, 2021>

1 (corrupt* or indulg* or absen* or brib* or kickback* or collu* or theft or steal* or illegal or crime or stole* or diver* or "pseudo-trial" or "pseudo trial" or fals* or fake or fraud* or counterfeit or nepotism or "informal pay" or "unnecessary refer*" or dishonest* or "informal practi*" or "illegal practi*" or "illicit practi*" or "speed up money" or "grease money" or "rent seek*" or embezzle* or extort* or skim* or gratuit* or "taint* loan*" or "bid rig*" or misappropriat* or conceal* or "price fix*" or "forc* labo?r" or inept* or "function abus*" or impunit* or squander* or larceny or cronyism or chisel* or patronage or favo?ritism or (abuse adj3 discretion) or (abuse adj3 power) or (crime adj3 "white-collar") or (crime adj3 "white collar") or launder* or neglig* or coerc* or deceiv* or deception or ghost* or blackmail* or clientelism or "private gain*" or (conflict* adj3 interest*)).mp. [mp=title, abstract, original title, name of substance word, subject heading word, floating sub-heading word, keyword heading word, organism supplementary concept word, protocol supplementary concept word, rare disease supplementary concept word, unique identifier, synonyms] 1899382

2 exp Fraud/ or exp Crime/ 141096

3 exp Deception/ 5158

4 exp Identity Theft/ or exp Theft/ or exp Medical Identity Theft/ 1726

5 exp Absenteeism/ 9361

6 (method* or measur* or detect* or surveill* or metric* or indicat* or survey* or interview* or scal* or audit* or monitor* or inquir* or "focus group*" or "discussion group*" or questionnaire* or report* or experiment* or trial* or test* or observ* or assess* or scor* or investig* or qualitative or quanti*).mp. [mp=title, abstract, original title, name of substance word, subject heading word, floating sub-heading word, keyword heading word, organism supplementary concept word, protocol supplementary concept word, rare disease supplementary concept word, unique identifier, synonyms] 22049205

7 exp Methods/ 700784

8 exp "Surveys and Questionnaires"/ 1108317

9 exp Interview/ 29795

10 exp Medical Audit/ or exp Clinical Audit/ 22686

11 exp Focus Groups/ 32461

12 exp "Weights and Measures"/ 244637

13 (((health* or "health-care" or frontline) adj3 (sector* or setting or cent* or facilit* or service* or system* or worker* or staff or provider* or unit* or personnel or deliver*)) or "healthcare" or health or hospital* or manag* or administrat* or "service provider*" or provider* or doctor* or physician* or nurs* or pharmac* or licen* or medic*).mp. [mp=title, abstract, original title, name of substance word, subject heading word, floating sub-heading word, keyword heading word, organism supplementary concept word, protocol supplementary concept word, rare disease supplementary concept word, unique identifier, synonyms] 12239812

14 exp Health Care Sector/ or exp Health Services/ or exp "Delivery of Health Care"/ or exp Health Expenditures/ 2913958

15 exp Health Resources/ 27805

16 exp Health Personnel/ 547893

17 exp Health Facilities/ 823003

18 exp Hospitals/ 288726

19 exp Health Services Accessibility/ 117594

20 exp Physicians/ 153165

21 exp Nurses/ 91370

22 exp "Organization and Administration"/ 1556498

23 exp Case Management/ or exp Decision Support Systems, Management/ or exp Total Quality Management/ or exp Health Information Management/ or exp Patient Care Management/ or exp Risk Management/ or exp Safety Management/ 1134243

24 exp Practice Patterns, Pharmacists'/ or exp Pharmacists/ or exp Pharmacy Service, Hospital/ 27304

25 exp Licensure, Medical/ or exp Licensure, Nursing/ or exp Licensure/ or exp Licensure, Dental/ or exp Licensure, Pharmacy/ or exp Licensure, Hospital/ 17905

26 exp Health Care Costs/ or exp Health Services Research/ or exp Health Equity/ or exp Public Health/ or exp Universal Health Care/ or exp Public Health Systems Research/ or exp Right to Health/ or exp Allied Health Personnel/ or exp Population Health/ or exp Insurance, Health, Reimbursement/ or exp Health/ or exp Health Facility Administration/ or exp Global Health/ or exp Health Care Reform/ or exp Primary Health Care/ 8812753

27 1 or 2 or 3 or 4 or 5 1999925

28 6 or 7 or 8 or 9 or 10 or 11 or 12 22152042

29 13 or 14 or 15 or 16 or 17 or 18 or 19 or 20 or 21 or 22 or 23 or 24 or 25 or 26 16827511

30 Developing Countries/ 77241

31 ((developing or less* developed or under developed or underdeveloped or middle income or low* income) adj (economy or economies)).ti,ab. 683

32 ((developing or less* developed or under developed or underdeveloped or middle income or low* income or underserved or under served or deprived or poor*) adj (countr* or nation? or population? or world)).ti,ab. 112014

33 (low* adj (gdp or gnp or gross domestic or gross national)).ti,ab. 287

34 (low adj3 middle adj3 countr*).ti,ab. 21149

35 (lmic or lmics or third world or lami countr*).ti,ab. 9323

36 transitional countr*.ti,ab. 171

37 global south.ti,ab. 550

38 "Democratic People's Republic of Korea"/ 251

39 (North Korea or (Democratic People* Republic adj2 Korea)).ti,ab. 455

40 Cambodia/ 3526

41 Cambodia.ti,ab. 4171

42 Indonesia/ 11700

43 (Indonesia or Dutch East Indies).ti,ab. 14323

44 (Kiribati or Gilbert Islands or Phoenix Islands or Line Islands).ti,ab. 260

45 Laos/ 2065

46 (Laos or (Lao adj1 Democratic Republic)).ti,ab. 2159

47 Micronesia/ 1216

48 Micronesia.ti,ab. 684

49 Mongolia/ 1904

50 Mongolia.ti,ab. 4490

51 Myanmar/ 2784

52 (Myanmar or Burma).ti,ab. 4669

53 Papua New Guinea/ 3578

54 (Papua New Guinea or German New Guinea or British New Guinea or Territory of Papua).ti,ab. 4679

55 Philippines/ 8800

56 (Philippines or Philippine Islands).ti,ab. 9154

57 Solomon Islands.ti,ab. 868

58 Timor-Leste/ 237

59 (Timor-Leste or East Timor or Portuguese Timor).ti,ab. 563

60 Vanuatu/ 381

61 (Vanuatu or New Hebrides).ti,ab. 745

62 Vietnam/ 13254

63 (Viet Nam or Vietnam or French Indochina).ti,ab. 16706

64 American Samoa/ 197

65 American Samoa.ti,ab. 377

66 exp China/ 223102

67 China.ti,ab. 214104

68 Fiji/ 1018

69 Fiji.ti,ab. 1901

70 Malaysia/ 16239

71 (Malaysia or Malayan Union or Malaya).ti,ab. 18017

72 Marshall Islands.ti,ab. 314

73 Nauru.ti,ab. 163

74 "Independent State of Samoa"/ 251

75 ((Samoa not American Samoa) or Western Samoa or Navigator Islands or Samoan Islands).ti,ab. 605

76 Thailand/ 27926

77 (Thailand or Siam).ti,ab. 28842

78 Tonga/ 256

79 Tonga.ti,ab. 459

80 (Tuvalu or Ellice Islands).ti,ab. 79

81 Melanesia/ 1119

82 Melanesia.ti,ab. 318

83 Polynesia/ 1937

84 Polynesia.ti,ab. 1379

85 Kyrgyzstan/ 1331

86 (Kyrgyzstan or Kyrgyz Republic or Kirghizia or Kirghiz).ti,ab. 1069

87 Moldova/ 716

88 Moldova.ti,ab. 566

89 Ukraine/ 16278

90 Ukraine.ti,ab. 5131

91 Uzbekistan/ 1936

92 Uzbekistan.ti,ab. 1190

93 Albania/ 900

94 Albania.ti,ab. 1143

95 Armenia/ 1472

96 Armenia.ti,ab. 1178

97 Azerbaijan/ 1247

98 Azerbaijan.ti,ab. 1485

99 "Republic of Belarus"/ 2108

100 (Belarus or Byelarus or Byelorussia or Belorussia).ti,ab. 1634

101 Bosnia-Herzegovina/ 2221

102 (Bosnia or Herzegovina).ti,ab. 2470

103 Bulgaria/ 6477

104 Bulgaria.ti,ab. 4414

105 "Georgia (Republic)"/ 1882

106 Georgia.ti,ab. not Georgia/ 6367

107 Kazakhstan/ 2810

108 (Kazakhstan or Kazakh).ti,ab. 3065

109 Kosovo/ 240

110 Kosovo.ti,ab. 974

111 Montenegro/ 241

112 Montenegro.ti,ab. 889

113 "Republic of North Macedonia"/ 599

114 North Macedonia.ti,ab. 104

115 Romania/ 10426

116 Romania.ti,ab. 6124

117 exp Russia/ 54717

118 "Russia (Pre-1917)"/ 5985

119 USSR/ 42807

120 (Russia or Russian Federation or USSR or Union of Soviet Socialist Republics or Soviet Union).ti,ab. 29844

121 Serbia/ 3375

122 Serbia/ 3375

123 Turkey/ 37166

124 (Turkey.ti,ab. not animal/) or (Anatolia or Asia Minor).ti,ab. 27784

125 Turkmenistan/ 582

126 Turkmenistan.ti,ab. 370

127 Tajikistan/ 773

128 Tajikistan.ti,ab. 647

129 Asia, Central/ 511

130 Asia, Northern/ 21

131 Central Asia.ti,ab. 2537

132 Haiti/ 3317

133 (Haiti or Hayti).ti,ab. 3197

134 Bolivia/ 2688

135 Bolivia.ti,ab. 3442

136 El Salvador/ 909

137 El Salvador.ti,ab. 1323

138 Honduras/ 1182

139 Honduras.ti,ab. 1874

140 Nicaragua/ 1544

141 Nicaragua.ti,ab. 1954

142 Argentina/ 16710

143 (Argentina or Argentine Republic).ti,ab. 17874

144 Belize/ 597

145 (Belize or British Honduras).ti,ab. 901

146 Brazil/ 102809

147 Brazil.ti,ab. 90966

148 Colombia/ 11410

149 Colombia.ti,ab. 13583

150 Costa Rica/ 3818

151 Costa Rica.ti,ab. 5148

152 Cuba/ 5178

153 Cuba.ti,ab. 4748

154 Dominica/ 103

155 Dominica.ti,ab. 506

156 Dominican Republic/ 1644

157 Dominican Republic.ti,ab. 2054

158 Ecuador/ 4121

159 Ecuador.ti,ab. 5081

160 Grenada/ 157

161 Grenada.ti,ab. 341

162 Guatemala/ 3123

163 Guatemala.ti,ab. 3767

164 Guyana/ 710

165 (Guyana or British Guiana).ti,ab. 1149

166 Jamaica/ 3498

167 Jamaica.ti,ab. 3341

168 Mexico/ 40619

169 (Mexico or United Mexican States).ti,ab. 45176

170 Paraguay/ 832

171 Paraguay.mp. 1823

172 Peru/ 9537

173 Peru.ti,ab. 11414

174 Saint Lucia/ 72

175 (St Lucia or Saint Lucia or Iyonala or Hewanorra).ti,ab. 352

176 "Saint Vincent and the Grenadines"/ 56

177 (Saint Vincent or St Vincent or Grenadines).ti,ab. 622

178 Suriname/ 966

179 (Suriname or Dutch Guiana).ti,ab. 636

180 Venezuela/ 5004

181 Venezuela.ti,ab. 5470

182 Djibouti/ 239

183 (Djibouti or French Somaliland).ti,ab. 407

184 Egypt/ 15790

185 Egypt.ti,ab. 15263

186 Morocco/ 6053

187 Morocco.ti,ab. 6082

188 Tunisia/ 8714

189 Tunisia.mp. 11009

190 (Gaza or West Bank or Palestine).ti,ab. 2728

191 Algeria/ 3242

192 Algeria.ti,ab. 3534

193 Iran/ 30500

194 (Iran or Persia).ti,ab. 42432

195 Iraq/ 4889

196 (Iraq or Mesopotamia).ti,ab. 7492

197 Jordan/ 4671

198 Jordan.ti,ab. 6928

199 Lebanon/ 4675

200 (Lebanon or Lebanese Republic).ti,ab. 4999

201 Libya/ 1159

202 Libya.ti,ab. 1336

203 Syria/ 2072

204 (Syria or Syrian Arab Republic).ti,ab. 2213

205 Yemen/ 1458

206 Yemen.ti,ab. 1954

207 Afghanistan/ 3423

208 Afghanistan.ti,ab. 6224

209 Nepal/ 9235

210 Nepal.ti,ab. 10890

211 Bangladesh/ 12191

212 Bangladesh.ti,ab. 15122

213 Bhutan/ 542

214 Bhutan.ti,ab. 860

215 exp India/ 109607

216 India.ti,ab. 107974

217 Pakistan/ 19584

218 Pakistan.ti,ab. 20715

219 Maldives.ti,ab. 375

220 Sri Lanka/ 6417

221 (Sri Lanka or Ceylon).ti,ab. 7515

222 Angola/ 1064

223 Angola.ti,ab. 1505

224 Cameroon/ 5859

225 (Cameroon or Kamerun or Cameroun).ti,ab. 7485

226 Cape Verde/ 228

227 (Cape Verde or Cabo Verde).ti,ab. 656

228 Comoros/ 331

229 (Comoros or Glorioso Islands or Mayotte).ti,ab. 615

230 Congo/ 1923

231 (Congo not ((Democratic Republic adj3 Congo) or congo red or crimean-congo)).ti,ab. 2720

232 Cote d'Ivoire/ 3264

233 (Cote d'Ivoire or Cote dIvoire or Ivory Coast).ti,ab. 4033

234 Eswatini/ 652

235 (eSwatini or Swaziland).ti,ab. 1003

236 Ghana/ 9172

237 (Ghana or Gold Coast).ti,ab. 12121

238 Kenya/ 17254

239 (Kenya or East Africa Protectorate).ti,ab. 19437

240 Lesotho/ 461

241 (Lesotho or Basutoland).ti,ab. 768

242 Mauritania/ 467

243 Mauritania.ti,ab. 668

244 Nigeria/ 30365

245 Nigeria.ti,ab. 30859

246 (Sao Tome adj2 Principe).ti,ab. 158

247 Senegal/ 5908

248 Senegal.ti,ab. 5939

249 Sudan/ 4891

250 (Sudan not South Sudan).ti,ab. 7758

251 Zambia/ 4846

252 (Zambia or Northern Rhodesia).ti,ab. 5700

253 Zimbabwe/ 6087

254 (Zimbabwe or Southern Rhodesia).ti,ab. 6047

255 Botswana/ 1939

256 (Botswana or Bechuanaland or Kalahari).ti,ab. 2760

257 Equatorial Guinea/ 283

258 (Equatorial Guinea or Spanish Guinea).ti,ab. 449

259 Gabon/ 1522

260 (Gabon or Gabonese Republic).ti,ab. 1833

261 Mauritius/ 588

262 (Mauritius or Agalega Islands).ti,ab. 1050

263 Namibia/ 1165

264 (Namibia or German South West Africa).ti,ab. 1682

265 South Africa/ 44696

266 (South Africa or Cape Colony or British Bechuanaland or Boer Republics or Zululand or Transvaal or Natalia Republic or Orange Free State).ti,ab. 37170

267 Benin/ 1692

268 (Benin or Dahomey).ti,ab. 3641

269 Burkina Faso/ 3452

270 (Burkina Faso or Burkina Fasso or Upper Volta).ti,ab. 4552

271 Burundi/ 664

272 (Burundi or Ruanda-Urundi).ti,ab. 980

273 Central African Republic/ 801

274 (Central African Republic or Ubangi-Shari).ti,ab. 1059

275 Chad/ 750

276 Chad.ti,ab. 1258

277 "Democratic Republic of the Congo"/ 4477

278 (((Democratic Republic or DR) adj2 Congo) or Congo-Kinshasa or Belgian Congo or Zaire or Congo Free State).ti,ab. 4814

279 Eritrea/ 376

280 Eritrea.ti,ab. 599

281 Ethiopia/ 14658

282 (Ethiopia or Abyssinia).ti,ab. 19202

283 Gambia/ 2503

284 Gambia.ti,ab. 2418

285 Guinea/ 1150

286 (Guinea not (New Guinea or Guinea Pig* or Guinea Fowl or Guinea-Bissau or Portuguese Guinea or Equatorial Guinea)).ti,ab. 2854

287 Guinea-Bissau/ 981

288 (Guinea-Bissau or Portuguese Guinea).ti,ab. 1083

289 Liberia/ 1254

290 Liberia.ti,ab. 1645

291 Madagascar/ 3633

292 (Madagascar or Malagasy Republic).ti,ab. 5095

293 Malawi/ 5824

294 (Malawi or Nyasaland).ti,ab. 7553

295 Mali/ 2475

296 Mali.ti,ab. 3730

297 Mozambique/ 2646

298 (Mozambique or Mocambique or Portuguese East Africa).ti,ab. 3930

299 Niger/ 1261

300 (Niger not (Aspergillus or Peptococcus or Schizothorax or Cruciferae or Gobius or Lasius or Agelastes or Melanosuchus or radish or Parastromateus or Orius or Apergillus or Parastromateus or Stomoxys)).ti,ab. 3635

301 Rwanda/ 2685

302 (Rwanda or Ruanda).ti,ab. 3392

303 Sierra Leone/ 1673

304 (Sierra Leone or Salone).ti,ab. 2451

305 Somalia/ 1690

306 (Somalia or Somaliland).ti,ab. 1600

307 South Sudan/ 187

308 South Sudan.ti,ab. 602

309 Tanzania/ 12220

310 (Tanzania or Tanganyika or Zanzibar).ti,ab. 14625

311 Togo/ 1194

312 (Togo or Togolese Republic or Togoland).ti,ab. 1577

313 Uganda/ 13182

314 Uganda.ti,ab. 15615

315 "africa south of the sahara"/ 11928

316 africa, central/ 1319

317 africa, eastern/ 4229

318 africa, southern/ 2476

319 africa, western/ 6055

320 ("Africa South of the Sahara" or sub-Saharan Africa or subSaharan Africa).ti,ab. 23779

321 Central Africa.ti,ab. 3316

322 Eastern Africa.ti,ab. 1096

323 Southern Africa.ti,ab. 4600

324 Western Africa.ti,ab. 908

325 or/30-324 [ALL LOW AND MIDDLE-INCOME COUNTRIES] 1622649

326 27 and 28 and 29 and 325 89534

327 limit 326 to (english language and full text and yr="2000 -Current") 5970

<https://ovidsp.ovid.com/ovidweb.cgi?T=JS&NEWS=N&PAGE=main&SHAREDSEARCHID=6wVU097DRLoiZNyv3S5G3zgc1k3vfVQ9oyleiDGHD2SmHRRIV5AGGTtGjirqFe5ZH>

Embase Classic+Embase <1947 to 2021 July 23>

1 (corrupt* or indulg* or absen* or brib* or kickback* or collu* or theft or steal* or illegal or crime or stole* or diver* or "pseudo-trial" or "pseudo trial" or fals* or fake or fraud* or counterfeit or nepotism or "informal pay" or "unnecessary refer*" or dishonest* or "informal practi*" or "illegal practi*" or "illicit practi*" or "speed up money" or "grease money" or "rent seek*" or embezzle* or extort* or skim* or gratuit* or "taint* loan*" or "bid rig*" or misappropriat* or conceal* or "price fix*" or "forc* labo?r" or inept* or "function abus*" or impunit* or squander* or larceny or cronyism or chisel* or patronage or favo?ritism or (abuse adj3 discretion) or (abuse adj3 power) or (crime adj3 "white-collar") or (crime adj3 "white collar") or launder* or neglig* or coerc* or deceiv* or deception or ghost* or blackmail* or clientelism or "private gain*" or (conflict* adj3 interest*)).mp. [mp=title, abstract, heading word, drug trade name, original title, device manufacturer, drug manufacturer, device trade name, keyword, floating subheading word, candidate term word] 2465659

2 exp forgery/ or exp fraud/ or exp crime/ 97899

3 exp deception/ 2892

4 exp theft/ or exp identity theft/ 3159

5 exp absenteeism/ 19840

6 (method* or measur* or detect* or surveill* or metric* or indicat* or survey* or interview* or scal* or audit* or monitor* or inquir* or "focus group*" or "discussion group*" or questionnaire* or report* or experiment* or trial* or test* or observ* or assess* or scor* or investig* or qualitative or quanti*).mp. [mp=title, abstract, heading word, drug trade name, original title, device manufacturer, drug manufacturer, device trade name, keyword, floating subheading word, candidate term word] 29234797

7 exp methodology/ 6536705

8 exp health survey/ 240708

9 exp short survey/ 578805

10 exp health care survey/ 19673

11 exp open ended questionnaire/ or exp questionnaire/ or exp structured questionnaire/ 785178

12 exp telephone interview/ or exp interview/ or exp structured interview/ or exp semi structured interview/ or exp unstructured interview/ 314392

13 exp quantitative analysis/ 355655

14 exp clinical audit/ 5976

15 exp monitor/ 64268

16 exp information processing/ 1979113

17 (((health* or "health-care" or frontline) adj3 (sector* or setting or cent* or facilit* or service* or system* or worker* or staff or provider* or unit* or personnel or deliver*)) or "healthcare" or health or hospital* or manag* or administrat* or "service provider*" or provider* or doctor* or physician* or nurs* or pharmac* or licen* or medic*).mp. [mp=title, abstract, heading word, drug trade name, original title, device manufacturer, drug manufacturer, device trade name, keyword, floating subheading word, candidate term word] 16330762

18 exp health care personnel management/ or exp integrated health care system/ or exp lay health worker/ or exp health care practice/ or exp tertiary health care/ or exp health service/ or exp health care facility/ or exp "health care facilities and services"/ or exp health care management/ or exp health care/ or exp health care delivery/ or exp health care system/ or exp health care planning/ or exp health workforce/ or exp health center/ or exp universal health care/ or exp population health/ or exp global health/ or exp health care access/ or exp primary health care/ or exp secondary health care/ or exp health care distribution/ or exp health care organization/ or exp universal health insurance/ or exp health care utilization/ or exp health services research/ or exp health care industry/ or exp health/ or exp health care personnel/ or exp public health/ 9073948

19 exp risk management/ or exp personnel management/ or exp management/ 1270085

20 exp licensing/ 97008

21 exp physician/ 862187

22 exp nurse/ 199451

23 exp pharmacist/ 85339

24 exp hospital/ or exp general hospital/ or exp hospital personnel management/ or exp hospital administrator/ or exp hospital organization/ 1338932

25 exp hospital billing/ or exp "billing and claims"/ 4490

26 1 or 2 or 3 or 4 or 5 2511179

27 6 or 7 or 8 or 9 or 10 or 11 or 12 or 13 or 14 or 15 or 16 29784303

28 exp health care personnel/ or exp medical personnel/ or exp administrative personnel/ or exp laboratory personnel/ or exp hospital personnel/ 1781191

29 17 or 18 or 19 or 20 or 21 or 22 or 23 or 24 or 25 or 28 18304125

30 developing country/ or low income country/ or middle income country/ 113122

31 ((developing or less* developed or under developed or underdeveloped or middle income or low* income) adj (economy or economies)).ti,ab. 853

32 ((developing or less* developed or under developed or underdeveloped or middle income or low* income or underserved or under served or deprived or poor*) adj (countr* or nation? or population? or world)).ti,ab. 142557

33 (low* adj (gdp or gnp or gross domestic or gross national)).ti,ab. 406

34 (low adj3 middle adj3 countr*).ti,ab. 24297

35 (lmic or lmics or third world or lami countr*).ti,ab. 11314

36 transitional countr*.ti,ab. 241

37 global south.ti,ab. 522

38 "Africa south of the Sahara"/ 15528

39 ("Africa South of the Sahara" or sub-Saharan Africa or subSaharan Africa).ti,ab. 28139

40 Central Africa.ti,ab. 3887

41 Eastern Africa.ti,ab. 1244

42 Southern Africa.ti,ab. 5134

43 Western Africa.ti,ab. 938

44 North Korea/ 619

45 (North Korea or (Democratic People* Republic adj2 Korea)).ti,ab. 510

46 Haiti/ 4481

47 (Haiti or Hayti).ti,ab. 4078

48 Afghanistan/ 6433

49 Afghanistan.ti,ab. 7499

50 Nepal/ 13514

51 Nepal.ti,ab. 13227

52 Syrian Arab Republic/ 2991

53 (Syria or Syrian Arab Republic).ti,ab. 2754

54 Yemen/ 2250

55 Yemen.ti,ab. 2316

56 Tajikistan/ 1061

57 Tajikistan.ti,ab. 803

58 Benin/ 2784

59 (Benin or Dahomey).ti,ab. 5072

60 Burkina Faso/ 4729

61 (Burkina Faso or Burkina Fasso or Upper Volta).ti,ab. 5438

62 Burundi/ 971

63 (Burundi or Ruanda-Urundi).ti,ab. 1098

64 Central African Republic/ 986

65 (Central African Republic or Ubangi-Shari).ti,ab. 1177

66 Chad/ 1063

67 Chad.ti,ab. 1593

68 Democratic Republic Congo/ 4676

69 (((Democratic Republic or DR) adj2 Congo) or Congo-Kinshasa or Belgian Congo or Zaire or Congo Free State).ti,ab. 5740

70 Eritrea/ 635

71 Eritrea.ti,ab. 730

72 Ethiopia/ 20942

73 (Ethiopia or Abyssinia).ti,ab. 20502

74 Gambia/ 2980

75 Gambia.ti,ab. 2722

76 Guinea/ 2942

77 (Guinea not (New Guinea or Guinea Pig* or Guinea Fowl or Guinea-Bissau or Portuguese Guinea or Equatorial Guinea)).ti,ab. 3398

78 Guinea-Bissau/ 1164

79 (Guinea-Bissau or Portuguese Guinea).ti,ab. 1200

80 Liberia/ 1931

81 Liberia.ti,ab. 1946

82 Madagascar/ 5011

83 (Madagascar or Malagasy Republic).ti,ab. 5769

84 Malawi/ 8337

85 (Malawi or Nyasaland).ti,ab. 8795

86 Mali/ 3803

87 Mali.ti,ab. 4708

88 Mozambique/ 4225

89 (Mozambique or Mocambique or Portuguese East Africa).ti,ab. 4592

90 Niger/ 2631

91 (Niger not (Aspergillus or Peptococcus or Schizothorax or Cruciferae or Gobius or Lasius or Agelastes or Melanosuchus or radish or Parastromateus or Orius or Apergillus or Parastromateus or Stomoxys)).ti,ab. 4548

92 Rwanda/ 4132

93 (Rwanda or Ruanda).ti,ab. 4113

94 Sierra Leone/ 2674

95 (Sierra Leone or Salone).ti,ab. 2834

96 Somalia/ 2160

97 (Somalia or Somaliland).ti,ab. 1777

98 south sudan/ 353

99 South Sudan.ti,ab. 694

100 Tanzania/ 16958

101 (Tanzania or Tanganyika or Zanzibar).ti,ab. 17207

102 Togo/ 1593

103 (Togo or Togolese Republic or Togoland).ti,ab. 1834

104 Uganda/ 20351

105 Uganda.ti,ab. 19499

106 Cambodia/ 5466

107 Cambodia.ti,ab. 5175

108 exp Indonesia/ 22426

109 (Indonesia or Dutch East Indies).ti,ab. 22550

110 kiribati/ 134

111 (Kiribati or Gilbert Islands or Phoenix Islands or Line Islands).ti,ab. 263

112 Laos/ 2390

113 (Laos or (Lao adj1 Democratic Republic)).ti,ab. 2368

114 exp "Federated States of Micronesia"/ 1042

115 Micronesia.ti,ab. 734

116 Mongolia/ 3485

117 Mongolia.ti,ab. 5448

118 Myanmar/ 4628

119 (Myanmar or Burma).ti,ab. 5427

120 Papua New Guinea/ 6992

121 (Papua New Guinea or German New Guinea or British New Guinea or Territory of Papua).ti,ab. 5156

122 Philippines/ 13187

123 (Philippines or Philippine Islands).ti,ab. 11615

124 solomon islands/ 587

125 Solomon Islands.ti,ab. 965

126 Timor-Leste/ 650

127 (Timor-Leste or East Timor or Portuguese Timor).ti,ab. 648

128 Vanuatu/ 501

129 (Vanuatu or New Hebrides).ti,ab. 798

130 Viet Nam/ 18852

131 (Viet Nam or Vietnam or French Indochina).ti,ab. 20148

132 Kyrgyzstan/ 1824

133 (Kyrgyzstan or Kyrgyz Republic or Kirghizia or Kirghiz).ti,ab. 1435

134 Moldova/ 1314

135 Moldova.ti,ab. 1038

136 exp Ukraine/ 17554

137 Ukraine.ti,ab. 7677

138 exp Uzbekistan/ 2570

139 Uzbekistan.ti,ab. 1845

140 Bolivia/ 3896

141 Bolivia.ti,ab. 4074

142 El Salvador/ 2187

143 El Salvador.ti,ab. 1700

144 Honduras/ 2215

145 Honduras.ti,ab. 2325

146 Nicaragua/ 2439

147 Nicaragua.ti,ab. 2427

148 Djibouti/ 384

149 (Djibouti or French Somaliland).ti,ab. 475

150 Egypt/ 23237

151 Egypt.ti,ab. 21259

152 Morocco/ 8712

153 Morocco.ti,ab. 8256

154 Tunisia/ 10846

155 Tunisia.mp. 13256

156 palestine/ 1892

157 (Gaza or West Bank or Palestine).ti,ab. 3362

158 Bangladesh/ 18278

159 Bangladesh.ti,ab. 18440

160 Bhutan/ 943

161 Bhutan.ti,ab. 948

162 exp India/ 168356

163 India.ti,ab. 152250

164 exp Pakistan/ 31656

165 Pakistan.ti,ab. 28699

166 Angola/ 1628

167 Angola.ti,ab. 1756

168 Cameroon/ 7907

169 (Cameroon or Kamerun or Cameroun).ti,ab. 8845

170 Cape Verde/ 427

171 (Cape Verde or Cabo Verde).ti,ab. 679

172 Comoros/ 369

173 (Comoros or Glorioso Islands or Mayotte).ti,ab. 655

174 Congo/ 4312

175 (Congo not ((Democratic Republic adj3 Congo) or congo red or crimean-congo)).ti,ab. 3645

176 Cote d'Ivoire/ 3567

177 (Cote d'Ivoire or Cote dIvoire or Ivory Coast).ti,ab. 4743

178 eswatini/ 172

179 (eSwatini or Swaziland).ti,ab. 1172

180 Ghana/ 13499

181 (Ghana or Gold Coast).ti,ab. 14428

182 Kenya/ 23777

183 (Kenya or East Africa Protectorate).ti,ab. 23271

184 Lesotho/ 806

185 (Lesotho or Basutoland).ti,ab. 887

186 Mauritania/ 705

187 Mauritania.ti,ab. 737

188 Nigeria/ 42301

189 Nigeria.ti,ab. 39909

190 "sao tome and principe"/ 90

191 (Sao Tome adj2 Principe).ti,ab. 167

192 Senegal/ 7428

193 Senegal.ti,ab. 7307

194 Sudan/ 7831

195 (Sudan not South Sudan).ti,ab. 10501

196 Zambia/ 7010

197 (Zambia or Northern Rhodesia).ti,ab. 6768

198 Zimbabwe/ 7590

199 (Zimbabwe or Southern Rhodesia).ti,ab. 6529

200 American Samoa/ 305

201 American Samoa.ti,ab. 441

202 china/ or guangxi/ or inner mongolia/ or macao/ or ningxia/ or tibet/ or xinjiang/ 244710

203 China.ti,ab. 251856

204 Fiji/ 1740

205 Fiji.ti,ab. 2447

206 exp Malaysia/ 26119

207 (Malaysia or Malayan Union or Malaya).ti,ab. 25441

208 marshall islands/ 181

209 Marshall Islands.ti,ab. 363

210 nauru/ 77

211 Nauru.ti,ab. 177

212 Samoa/ 707

213 ((Samoa not American Samoa) or Western Samoa or Navigator Islands or Samoan Islands).ti,ab. 701

214 Thailand/ 37379

215 (Thailand or Siam).ti,ab. 35724

216 Tonga/ 392

217 Tonga.ti,ab. 506

218 tuvalu/ 55

219 (Tuvalu or Ellice Islands).ti,ab. 79

220 Albania/ 1993

221 Albania.ti,ab. 1827

222 Armenia/ 2210

223 Armenia.ti,ab. 1791

224 exp Azerbaijan/ 2016

225 Azerbaijan.ti,ab. 2187

226 Belarus/ 3014

227 (Belarus or Byelarus or Byelorussia or Belorussia).ti,ab. 2628

228 exp "Bosnia and Herzegovina"/ 2957

229 (Bosnia or Herzegovina).ti,ab. 3194

230 Bulgaria/ 10319

231 Bulgaria.ti,ab. 7324

232 exp "Georgia (republic)"/ 2144

233 Georgia.ti,ab. not "georgia (u.s.)"/ 12361

234 Kazakhstan/ 4451

235 (Kazakhstan or Kazakh).ti,ab. 4652

236 Kosovo/ 603

237 Kosovo.ti,ab. 1340

238 "Montenegro (republic)"/ 810

239 Montenegro.ti,ab. 1246

240 "republic of north macedonia"/ 85

241 North Macedonia.ti,ab. 129

242 Romania/ 15606

243 Romania.ti,ab. 10378

244 exp Russian Federation/ 67440

245 ussr/ 48198

246 (Russia or Russian Federation or USSR or Union of Soviet Socialist Republics or Soviet Union).ti,ab. 40653

247 exp Serbia/ 6377

248 Serbia.ti,ab. 7418

249 "Turkey (republic)"/ 39624

250 (Turkey.ti,ab. not "Turkey (bird)"/) or (Anatolia or Asia Minor).ti,ab. 47394

251 Turkmenistan/ 653

252 Turkmenistan.ti,ab. 410

253 Argentina/ 24784

254 (Argentina or Argentine Republic).ti,ab. 24520

255 Belize/ 851

256 (Belize or British Honduras).ti,ab. 1026

257 exp Brazil/ 128231

258 Brazil.ti,ab. 113366

259 Colombia/ 22415

260 Colombia.ti,ab. 19367

261 Costa Rica/ 5249

262 Costa Rica.ti,ab. 5570

263 Cuba/ 7363

264 Cuba.ti,ab. 6400

265 Dominica/ 215

266 Dominica.ti,ab. 578

267 Dominican Republic/ 2795

268 Dominican Republic.ti,ab. 2603

269 Ecuador/ 6298

270 Ecuador.ti,ab. 6388

271 Grenada/ 303

272 Grenada.ti,ab. 418

273 Guatemala/ 4981

274 Guatemala.ti,ab. 4724

275 Guyana/ 1142

276 (Guyana or British Guiana).ti,ab. 1484

277 Jamaica/ 4556

278 Jamaica.ti,ab. 4224

279 exp Mexico/ 52800

280 (Mexico or United Mexican States).ti,ab. 56994

281 Paraguay/ 1655

282 Paraguay.mp. 2445

283 Peru/ 14040

284 Peru.ti,ab. 14827

285 Saint Lucia/ 137

286 (St Lucia or Saint Lucia or Iyonala or Hewanorra).ti,ab. 410

287 "Saint Vincent and the Grenadines"/ 81

288 (Saint Vincent or St Vincent or Grenadines).ti,ab. 925

289 Suriname/ 1335

290 (Suriname or Dutch Guiana).ti,ab. 798

291 Venezuela/ 7136

292 Venezuela.ti,ab. 6946

293 Algeria/ 5530

294 Algeria.ti,ab. 5584

295 Iran/ 56793

296 (Iran or Persia).ti,ab. 59705

297 exp Iraq/ 10789

298 (Iraq or Mesopotamia).ti,ab. 12242

299 Jordan/ 7754

300 Jordan.ti,ab. 9176

301 Lebanon/ 6669

302 (Lebanon or Lebanese Republic).ti,ab. 6337

303 Libyan Arab Jamahiriya/ 1837

304 libya.ti,ab. 1649

305 maldives/ 346

306 Maldives.ti,ab. 410

307 Sri Lanka/ 9607

308 (Sri Lanka or Ceylon).ti,ab. 9485

309 Botswana/ 2992

310 (Botswana or Bechuanaland or Kalahari).ti,ab. 3279

311 Equatorial Guinea/ 513

312 (Equatorial Guinea or Spanish Guinea).ti,ab. 601

313 Gabon/ 1879

314 (Gabon or Gabonese Republic).ti,ab. 2078

315 Mauritius/ 995

316 (Mauritius or Agalega Islands).ti,ab. 1141

317 Namibia/ 1791

318 (Namibia or German South West Africa).ti,ab. 1860

319 South Africa/ 56406

320 (South Africa or Cape Colony or British Bechuanaland or Boer Republics or Zululand or Transvaal or Natalia Republic or Orange Free State).ti,ab. 45291

321 or/30-320 [ALL LOW AND MIDDLE-INCOME COUNTRIES] 1931039

322 26 and 27 and 29 and 321 80214

323 limit 322 to (full text and english language and yr="2000 -Current") 4733

<https://ovidsp.ovid.com/ovidweb.cgi?T=JS&NEWS=N&PAGE=main&SHAREDSEARCHID=6Yj1WJhzVRzTIVRlENq8MBraFLlGpi6nnQ30XdKDjSR82sarILr0Mnb9RwsXoX7G2>

Econlit <1886 to July 15, 2021>

1 (corrupt* or indulg* or absen* or brib* or kickback* or collu* or theft or steal* or illegal or crime or stole* or diver* or "pseudo-trial" or "pseudo trial" or fals* or fake or fraud* or counterfeit or nepotism or "informal pay" or "unnecessary refer*" or dishonest* or "informal practi*" or "illegal practi*" or "illicit practi*" or "speed up money" or "grease money" or "rent seek*" or embezzle* or extort* or skim* or gratuit* or "taint* loan*" or "bid rig*" or misappropriat* or conceal* or "price fix*" or "forc* labo?r" or inept* or "function abus*" or impunit* or squander* or larceny or cronyism or chisel* or patronage or favo?ritism or (abuse adj3 discretion) or (abuse adj3 power) or (crime adj3 "white-collar") or (crime adj3 "white collar") or launder* or neglig* or coerc* or deceiv* or deception or ghost* or blackmail* or clientelism or "private gain*" or (conflict* adj3 interest*)).mp. [mp=heading words, abstract, title, country as subject] 211253

2 (method* or measur* or detect* or surveill* or metric* or indicat* or survey* or interview* or scal* or audit* or monitor* or inquir* or "focus group*" or "discussion group*" or questionnaire* or report* or experiment* or trial* or test* or observ* or assess* or scor* or investig* or qualitative or quanti*).mp. [mp=heading words, abstract, title, country as subject] 762104

3 (((health* or "health-care" or frontline) adj3 (sector* or setting or cent* or facilit* or service* or system* or worker* or staff or provider* or unit* or personnel or deliver*)) or "healthcare" or health or hospital* or manag* or administrat* or "service provider*" or provider* or doctor* or physician* or nurs* or pharmac* or licen* or medic*).mp. [mp=heading words, abstract, title, country as subject] 397573

4 developing country/ or low income country/ or middle income country/ 0

5 ((developing or less* developed or under developed or underdeveloped or middle income or low* income) adj (economy or economies)).ti,ab. 4983

6 ((developing or less* developed or under developed or underdeveloped or middle income or low* income or underserved or under served or deprived or poor*) adj (countr* or nation? or population? or world)).ti,ab. 44232

7 (low* adj (gdp or gnp or gross domestic or gross national)).ti,ab. 198

8 (low adj3 middle adj3 countr*).ti,ab. 1166

9 (lmic or lmics or third world or lami countr*).ti,ab. 1898

10 transitional countr*.ti,ab. 223

11 global south.ti,ab. 743

12 "Africa south of the Sahara"/ 0

13 ("Africa South of the Sahara" or sub-Saharan Africa or subSaharan Africa).ti,ab. 5384

14 Central Africa.ti,ab. 218

15 Eastern Africa.ti,ab. 98

16 Southern Africa.ti,ab. 977

17 Western Africa.ti,ab. 49

18 North Korea/ 0

19 (North Korea or (Democratic People* Republic adj2 Korea)).ti,ab. 357

20 Haiti/ 0

21 (Haiti or Hayti).ti,ab. 331

22 Afghanistan/ 0

23 Afghanistan.ti,ab. 624

24 Nepal/ 0

25 Nepal.ti,ab. 1288

26 Syrian Arab Republic/ 0

27 (Syria or Syrian Arab Republic).ti,ab. 395

28 Yemen/ 0

29 Yemen.ti,ab. 205

30 Tajikistan/ 0

31 Tajikistan.ti,ab. 265

32 Benin/ 0

33 (Benin or Dahomey).ti,ab. 385

34 Burkina Faso/ 0

35 (Burkina Faso or Burkina Fasso or Upper Volta).ti,ab. 724

36 Burundi/ 0

37 (Burundi or Ruanda-Urundi).ti,ab. 217

38 Central African Republic/ 0

39 (Central African Republic or Ubangi-Shari).ti,ab. 53

40 Chad/ 0

41 Chad.ti,ab. 166

42 Democratic Republic Congo/ 0

43 (((Democratic Republic or DR) adj2 Congo) or Congo-Kinshasa or Belgian Congo or Zaire or Congo Free State).ti,ab. 374

44 Eritrea/ 0

45 Eritrea.ti,ab. 107

46 Ethiopia/ 0

47 (Ethiopia or Abyssinia).ti,ab. 2261

48 Gambia/ 0

49 Gambia.ti,ab. 178

50 Guinea/ 0

51 (Guinea not (New Guinea or Guinea Pig* or Guinea Fowl or Guinea-Bissau or Portuguese Guinea or Equatorial Guinea)).ti,ab. 176

52 Guinea-Bissau/ 0

53 (Guinea-Bissau or Portuguese Guinea).ti,ab. 76

54 Liberia/ 0

55 Liberia.ti,ab. 258

56 Madagascar/ 0

57 (Madagascar or Malagasy Republic).ti,ab. 524

58 Malawi/ 0

59 (Malawi or Nyasaland).ti,ab. 1236

60 Mali/ 0

61 Mali.ti,ab. 645

62 Mozambique/ 0

63 (Mozambique or Mocambique or Portuguese East Africa).ti,ab. 942

64 Niger/ 0

65 (Niger not (Aspergillus or Peptococcus or Schizothorax or Cruciferae or Gobius or Lasius or Agelastes or Melanosuchus or radish or Parastromateus or Orius or Apergillus or Parastromateus or Stomoxys)).ti,ab. 494

66 Rwanda/ 0

67 (Rwanda or Ruanda).ti,ab. 668

68 Sierra Leone/ 0

69 (Sierra Leone or Salone).ti,ab. 407

70 Somalia/ 0

71 (Somalia or Somaliland).ti,ab. 205

72 south sudan/ 0

73 South Sudan.ti,ab. 80

74 Tanzania/ 0

75 (Tanzania or Tanganyika or Zanzibar).ti,ab. 2366

76 Togo/ 0

77 (Togo or Togolese Republic or Togoland).ti,ab. 169

78 Uganda/ 0

79 Uganda.ti,ab. 1997

80 Cambodia/ 0

81 Cambodia.ti,ab. 715

82 exp Indonesia/ 0

83 (Indonesia or Dutch East Indies).ti,ab. 6794

84 kiribati/ 0

85 (Kiribati or Gilbert Islands or Phoenix Islands or Line Islands).ti,ab. 47

86 Laos/ 0

87 (Laos or (Lao adj1 Democratic Republic)).ti,ab. 308

88 exp "Federated States of Micronesia"/ 0

89 Micronesia.ti,ab. 32

90 Mongolia/ 0

91 Mongolia.ti,ab. 407

92 Myanmar/ 0

93 (Myanmar or Burma).ti,ab. 544

94 Papua New Guinea/ 0

95 (Papua New Guinea or German New Guinea or British New Guinea or Territory of Papua).ti,ab. 532

96 Philippines/ 0

97 (Philippines or Philippine Islands).ti,ab. 3629

98 solomon islands/ 0

99 Solomon Islands.ti,ab. 153

100 Timor-Leste/ 0

101 (Timor-Leste or East Timor or Portuguese Timor).ti,ab. 163

102 Vanuatu/ 0

103 (Vanuatu or New Hebrides).ti,ab. 120

104 Viet Nam/ 0

105 (Viet Nam or Vietnam or French Indochina).ti,ab. 3900

106 Kyrgyzstan/ 0

107 (Kyrgyzstan or Kyrgyz Republic or Kirghizia or Kirghiz).ti,ab. 470

108 Moldova/ 0

109 Moldova.ti,ab. 322

110 exp Ukraine/ 0

111 Ukraine.ti,ab. 2780

112 exp Uzbekistan/ 0

113 Uzbekistan.ti,ab. 377

114 Bolivia/ 0

115 Bolivia.ti,ab. 1115

116 El Salvador/ 0

117 El Salvador.ti,ab. 481

118 Honduras/ 0

119 Honduras.ti,ab. 454

120 Nicaragua/ 0

121 Nicaragua.ti,ab. 679

122 Djibouti/ 0

123 (Djibouti or French Somaliland).ti,ab. 47

124 Egypt/ 0

125 Egypt.ti,ab. 2231

126 Morocco/ 0

127 Morocco.ti,ab. 1202

128 Tunisia/ 0

129 Tunisia.mp. 1943

130 palestine/ 0

131 (Gaza or West Bank or Palestine).ti,ab. 482

132 Bangladesh/ 0

133 Bangladesh.ti,ab. 4276

134 Bhutan/ 0

135 Bhutan.ti,ab. 149

136 exp India/ 0

137 India.ti,ab. 24606

138 exp Pakistan/ 0

139 Pakistan.ti,ab. 6905

140 Angola/ 0

141 Angola.ti,ab. 288

142 Cameroon/ 0

143 (Cameroon or Kamerun or Cameroun).ti,ab. 868

144 Cape Verde/ 0

145 (Cape Verde or Cabo Verde).ti,ab. 94

146 Comoros/ 0

147 (Comoros or Glorioso Islands or Mayotte).ti,ab. 20

148 Congo/ 0

149 (Congo not ((Democratic Republic adj3 Congo) or congo red or crimean-congo)).ti,ab. 221

150 Cote d'Ivoire/ 0

151 (Cote d'Ivoire or Cote dIvoire or Ivory Coast).ti,ab. 801

152 eswatini/ 0

153 (eSwatini or Swaziland).ti,ab. 182

154 Ghana/ 0

155 (Ghana or Gold Coast).ti,ab. 3331

156 Kenya/ 0

157 (Kenya or East Africa Protectorate).ti,ab. 3160

158 Lesotho/ 0

159 (Lesotho or Basutoland).ti,ab. 271

160 Mauritania/ 0

161 Mauritania.ti,ab. 97

162 Nigeria/ 0

163 Nigeria.ti,ab. 4133

164 "sao tome and principe"/ 0

165 (Sao Tome adj2 Principe).ti,ab. 39

166 Senegal/ 0

167 Senegal.ti,ab. 857

168 Sudan/ 0

169 (Sudan not South Sudan).ti,ab. 596

170 Zambia/ 0

171 (Zambia or Northern Rhodesia).ti,ab. 1148

172 Zimbabwe/ 0

173 (Zimbabwe or Southern Rhodesia).ti,ab. 1297

174 American Samoa/ 0

175 American Samoa.ti,ab. 14

176 china/ or guangxi/ or inner mongolia/ or macao/ or ningxia/ or tibet/ or xinjiang/ 0

177 China.ti,ab. 41140

178 Fiji/ 0

179 Fiji.ti,ab. 494

180 exp Malaysia/ 0

181 (Malaysia or Malayan Union or Malaya).ti,ab. 5405

182 marshall islands/ 0

183 Marshall Islands.ti,ab. 19

184 nauru/ 0

185 Nauru.ti,ab. 21

186 Samoa/ 0

187 ((Samoa not American Samoa) or Western Samoa or Navigator Islands or Samoan Islands).ti,ab. 92

188 Thailand/ 0

189 (Thailand or Siam).ti,ab. 4690

190 Tonga/ 0

191 Tonga.ti,ab. 107

192 tuvalu/ 0

193 (Tuvalu or Ellice Islands).ti,ab. 31

194 Albania/ 0

195 Albania.ti,ab. 695

196 Armenia/ 0

197 Armenia.ti,ab. 352

198 exp Azerbaijan/ 0

199 Azerbaijan.ti,ab. 381

200 Belarus/ 0

201 (Belarus or Byelarus or Byelorussia or Belorussia).ti,ab. 410

202 exp "Bosnia and Herzegovina"/ 0

203 (Bosnia or Herzegovina).ti,ab. 837

204 Bulgaria/ 0

205 Bulgaria.ti,ab. 2436

206 exp "Georgia (republic)"/ 0

207 Georgia.ti,ab. not "georgia (u.s.)"/ 0

208 Kazakhstan/ 0

209 (Kazakhstan or Kazakh).ti,ab. 1000

210 Kosovo/ 0

211 Kosovo.ti,ab. 305

212 "Montenegro (republic)"/ 0

213 Montenegro.ti,ab. 328

214 "republic of north macedonia"/ 0

215 North Macedonia.ti,ab. 27

216 Romania/ 0

217 Romania.ti,ab. 3400

218 exp Russian Federation/ 0

219 ussr/ 0

220 (Russia or Russian Federation or USSR or Union of Soviet Socialist Republics or Soviet Union).ti,ab. 12057

221 exp Serbia/ 0

222 Serbia.ti,ab. 1352

223 "Turkey (republic)"/ 0

224 (Turkey.ti,ab. not "Turkey (bird)"/) or (Anatolia or Asia Minor).ti,ab. 0

225 Turkmenistan/ 0

226 Turkmenistan.ti,ab. 134

227 Argentina/ 0

228 (Argentina or Argentine Republic).ti,ab. 5101

229 Belize/ 0

230 (Belize or British Honduras).ti,ab. 81

231 exp Brazil/ 0

232 Brazil.ti,ab. 11208

233 Colombia/ 0

234 Colombia.ti,ab. 4784

235 Costa Rica/ 0

236 Costa Rica.ti,ab. 1036

237 Cuba/ 0

238 Cuba.ti,ab. 704

239 Dominica/ 0

240 Dominica.ti,ab. 38

241 Dominican Republic/ 0

242 Dominican Republic.ti,ab. 502

243 Ecuador/ 0

244 Ecuador.ti,ab. 1178

245 Grenada/ 0

246 Grenada.ti,ab. 72

247 Guatemala/ 0

248 Guatemala.ti,ab. 640

249 Guyana/ 0

250 (Guyana or British Guiana).ti,ab. 272

251 Jamaica/ 0

252 Jamaica.ti,ab. 927

253 exp Mexico/ 0

254 (Mexico or United Mexican States).ti,ab. 11988

255 Paraguay/ 0

256 Paraguay.mp. 448

257 Peru/ 0

258 Peru.ti,ab. 2352

259 Saint Lucia/ 0

260 (St Lucia or Saint Lucia or Iyonala or Hewanorra).ti,ab. 52

261 "Saint Vincent and the Grenadines"/ 0

262 (Saint Vincent or St Vincent or Grenadines).ti,ab. 42

263 Suriname/ 0

264 (Suriname or Dutch Guiana).ti,ab. 56

265 Venezuela/ 0

266 Venezuela.ti,ab. 1070

267 Algeria/ 0

268 Algeria.ti,ab. 634

269 Iran/ 0

270 (Iran or Persia).ti,ab. 3011

271 exp Iraq/ 0

272 (Iraq or Mesopotamia).ti,ab. 980

273 Jordan/ 0

274 Jordan.ti,ab. 1123

275 Lebanon/ 0

276 (Lebanon or Lebanese Republic).ti,ab. 540

277 Libyan Arab Jamahiriya/ 0

278 libya.ti,ab. 241

279 maldives/ 0

280 Maldives.ti,ab. 89

281 Sri Lanka/ 0

282 (Sri Lanka or Ceylon).ti,ab. 1766

283 Botswana/ 0

284 (Botswana or Bechuanaland or Kalahari).ti,ab. 670

285 Equatorial Guinea/ 0

286 (Equatorial Guinea or Spanish Guinea).ti,ab. 33

287 Gabon/ 0

288 (Gabon or Gabonese Republic).ti,ab. 105

289 Mauritius/ 0

290 (Mauritius or Agalega Islands).ti,ab. 482

291 Namibia/ 0

292 (Namibia or German South West Africa).ti,ab. 334

293 South Africa/ 0

294 (South Africa or Cape Colony or British Bechuanaland or Boer Republics or Zululand or Transvaal or Natalia Republic or Orange Free State).ti,ab. 7826

295 or/4-294 [ALL LOW AND MIDDLE-INCOME COUNTRIES] 228443

296 1 and 2 and 3 and 295 9267

297 limit 296 to (full text and yr="2000 -Current" and english) 146

<https://ovidsp.ovid.com/ovidweb.cgi?T=JS&NEWS=N&PAGE=main&SHAREDSEARCHID=2aFUrOftjGq6UXQip0VaE1VPvGex2RjiQamDBvPAlh4gXrTxT68KW4OjqsFWigN9o>

Global Health <1910 to 2021 Week 29>

1 (corrupt* or indulg* or absen* or brib* or kickback* or collu* or theft or steal* or illegal or crime or stole* or diver* or "pseudo-trial" or "pseudo trial" or fals* or fake or fraud* or counterfeit or nepotism or "informal pay" or "unnecessary refer*" or dishonest* or "informal practi*" or "illegal practi*" or "illicit practi*" or "speed up money" or "grease money" or "rent seek*" or embezzle* or extort* or skim* or gratuit* or "taint* loan*" or "bid rig*" or misappropriat* or conceal* or "price fix*" or "forc* labo?r" or inept* or "function abus*" or impunit* or squander* or larceny or cronyism or chisel* or patronage or favo?ritism or (abuse adj3 discretion) or (abuse adj3 power) or (crime adj3 "white-collar") or (crime adj3 "white collar") or launder* or neglig* or coerc* or deceiv* or deception or ghost* or blackmail* or clientelism or "private gain*" or (conflict* adj3 interest*)).mp. [mp=abstract, title, original title, broad terms, heading words, identifiers, cabicodes] 289655

2 exp corruption/ 143

3 exp theft/ or exp employee theft/ 47

4 exp crime/ 6967

5 (method* or measur* or detect* or surveill* or metric* or indicat* or survey* or interview* or scal* or audit* or monitor* or inquir* or "focus group*" or "discussion group*" or questionnaire* or report* or experiment* or trial* or test* or observ* or assess* or scor* or investig* or qualitative or quanti*).mp. [mp=abstract, title, original title, broad terms, heading words, identifiers, cabicodes] 3475048

6 exp methodology/ 111954

7 exp monitoring/ 48774

8 exp auditing/ 428

9 exp interviews/ 2746

10 exp questionnaires/ 41403

11 exp surveys/ 117852

12 exp trials/ 70775

13 exp detection/ 72515

14 exp surveillance/ 50655

15 (((health* or "health-care" or frontline) adj3 (sector* or setting or cent* or facilit* or service* or system* or worker* or staff or provider* or unit* or personnel or deliver*)) or "healthcare" or health or hospital* or manag* or administrat* or "service provider*" or provider* or doctor* or physician* or nurs* or pharmac* or licen* or medic*).mp. [mp=abstract, title, original title, broad terms, heading words, identifiers, cabicodes] 1870683

16 exp health services/ or exp health care/ or exp universal health coverage/ or exp health inequalities/ or exp community health/ or exp health clinics/ or exp primary health care/ or exp health care utilization/ or exp health claims/ or exp health insurance/ or exp community health services/ or exp health care costs/ or exp public health services/ or exp health centres/ or exp health care workers/ or exp health/ or exp community health workers/ or exp public health/ or exp dental health/ 522469

17 exp nurses/ 10452

18 exp physicians/ 38998

19 exp personnel management/ or exp hospital personnel/ or exp personnel/ 31463

20 exp management/ 72949

21 exp administration/ 2293

22 exp resources/ 880

23 exp hospitals/ 59205

24 exp medicine/ 95147

25 licences.sh. 780

26 exp resource management/ 58450

27 exp medical services/ 16672

28 exp pharmacy/ 2094

29 1 or 2 or 3 or 4 294143

30 5 or 6 or 7 or 8 or 9 or 10 or 11 or 12 or 13 or 14 3476525

31 15 or 16 or 17 or 18 or 19 or 20 or 21 or 22 or 23 or 24 or 25 or 26 or 27 or 28 1917999

32 developing countries/ 1072185

33 least developed countries/ 137989

34 Threshold Countries/ 334597

35 ((developing or less* developed or under developed or underdeveloped or middle income or low* income) adj (economy or economies)).ti,ab. 363

36 ((developing or less* developed or under developed or underdeveloped or middle income or low* income or underserved or under served or deprived or poor*) adj (countr* or nation? or population? or world)).ti,ab. 63172

37 (low* adj (gdp or gnp or gross domestic or gross national)).ti,ab. 86

38 (low adj3 middle adj3 countr*).ti,ab. 11203

39 (lmic or lmics or third world or lami countr*).ti,ab. 4509

40 transitional countr*.ti,ab. 105

41 global south.ti,ab. 298

42 korea democratic people's republic/ 343

43 (North Korea or (Democratic People* Republic adj2 Korea)).ti,ab. 241

44 cambodia/ 3394

45 Cambodia.ti,ab. 3213

46 exp indonesia/ 17113

47 (Indonesia or Dutch East Indies).ti,ab. 11154

48 kiribati/ 188

49 (Kiribati or Gilbert Islands or Phoenix Islands or Line Islands).ti,ab. 173

50 laos/ 1980

51 (Laos or (Lao adj1 Democratic Republic)).ti,ab. 1504

52 "federated states of micronesia"/ 191

53 Micronesia.ti,ab. 363

54 mongolia/ 1536

55 Mongolia.ti,ab. 2276

56 myanmar/ 4382

57 (Myanmar or Burma).ti,ab. 4437

58 papua new guinea/ 3789

59 (Papua New Guinea or German New Guinea or British New Guinea or Territory of Papua).ti,ab. 3579

60 exp philippines/ 9097

61 (Philippines or Philippine Islands).ti,ab. 7904

62 solomon islands/ 868

63 Solomon Islands.ti,ab. 835

64 east timor/ 316

65 (Timor-Leste or East Timor or Portuguese Timor).ti,ab. 405

66 vanuatu/ 635

67 (Vanuatu or New Hebrides).ti,ab. 666

68 vietnam/ 10520

69 (Viet Nam or Vietnam or French Indochina).ti,ab. 9255

70 american samoa/ 269

71 American Samoa.ti,ab. 276

72 exp china/ 202641

73 China.ti,ab. 103321

74 fiji/ 1340

75 Fiji.ti,ab. 1331

76 exp malaysia/ 18052

77 (Malaysia or Malayan Union or Malaya).ti,ab. 13798

78 marshall islands/ 178

79 Marshall Islands.ti,ab. 165

80 nauru/ 144

81 Nauru.ti,ab. 147

82 samoa/ 473

83 ((Samoa not American Samoa) or Western Samoa or Navigator Islands or Samoan Islands).ti,ab. 648

84 thailand/ 25862

85 (Thailand or Siam).ti,ab. 20568

86 tonga/ 335

87 Tonga.ti,ab. 358

88 tuvalu/ 108

89 (Tuvalu or Ellice Islands).ti,ab. 95

90 melanesia/ 8204

91 Melanesia.ti,ab. 179

92 polynesia/ 5905

93 Polynesia.ti,ab. 806

94 kyrgyzstan/ 754

95 (Kyrgyzstan or Kyrgyz Republic or Kirghizia or Kirghiz).ti,ab. 511

96 moldova/ 741

97 Moldova.ti,ab. 431

98 ukraine/ 3813

99 Ukraine.ti,ab. 2555

100 uzbekistan/ 1880

101 Uzbekistan.ti,ab. 1063

102 albania/ 1192

103 Albania.ti,ab. 1016

104 armenia/ 839

105 Armenia.ti,ab. 708

106 azerbaijan/ 1327

107 Azerbaijan.ti,ab. 1151

108 belarus/ 1124

109 (Belarus or Byelarus or Byelorussia or Belorussia).ti,ab. 1000

110 bosnia-hercegovina/ 1791

111 (Bosnia or Herzegovina).ti,ab. 1286

112 bulgaria/ 5419

113 Bulgaria.ti,ab. 3589

114 "republic of georgia"/ 1169

115 Georgia.ti,ab. not Georgia/ 1571

116 kazakhstan/ 2896

117 (Kazakhstan or Kazakh).ti,ab. 2483

118 kosovo/ 415

119 Kosovo.ti,ab. 535

120 montenegro/ 305

121 Montenegro.ti,ab. 788

122 "republic of north macedonia"/ 89

123 North Macedonia.ti,ab. 35

124 romania/ 9701

125 Romania.ti,ab. 4694

126 exp russia/ 19066

127 exp ussr/ 21061

128 (Russia or Russian Federation or USSR or Union of Soviet Socialist Republics or Soviet Union).ti,ab. 18508

129 exp serbia/ or "serbia and montenegro"/ 4303

130 serbia.ti,ab. 2993

131 turkey/ 36526

132 (Turkey.ti,ab. not animals/) or (Anatolia or Asia Minor).ti,ab. 6425

133 turkmenistan/ 652

134 Turkmenistan.ti,ab. 294

135 tajikistan/ 818

136 Tajikistan.ti,ab. 443

137 central asia/ 14666

138 Central Asia.ti,ab. 1569

139 haiti/ 2267

140 (Haiti or Hayti).ti,ab. 2266

141 bolivia/ 2851

142 Bolivia.ti,ab. 2645

143 el salvador/ 1414

144 El Salvador.ti,ab. 1065

145 honduras/ 1480

146 Honduras.ti,ab. 1530

147 nicaragua/ 1491

148 Nicaragua.ti,ab. 1410

149 argentina/ 14871

150 (Argentina or Argentine Republic).ti,ab. 10740

151 belize/ 595

152 (Belize or British Honduras).ti,ab. 621

153 exp brazil/ 102114

154 Brazil.ti,ab. 70981

155 colombia/ 11611

156 Colombia.ti,ab. 9986

157 costa rica/ 3171

158 Costa Rica.ti,ab. 3034

159 cuba/ 5584

160 Cuba.ti,ab. 4376

161 dominica/ 191

162 Dominica.ti,ab. 337

163 dominican republic/ 1165

164 Dominican Republic.ti,ab. 1224

165 exp ecuador/ 3643

166 Ecuador.ti,ab. 3397

167 grenada/ 296

168 Grenada.ti,ab. 302

169 guatemala/ 4045

170 Guatemala.ti,ab. 3593

171 guyana/ 1172

172 (Guyana or British Guiana).ti,ab. 1257

173 jamaica/ 2871

174 Jamaica.ti,ab. 2683

175 mexico/ 25831

176 (Mexico or United Mexican States).ti,ab. 23701

177 paraguay/ 1373

178 Paraguay.mp. 1608

179 peru/ 9134

180 Peru.ti,ab. 7651

181 saint lucia/ 411

182 (St Lucia or Saint Lucia or Iyonala or Hewanorra).ti,ab. 482

183 grenadines/ or exp "saint vincent and the grenadines"/ 178

184 (Saint Vincent or St Vincent or Grenadines).ti,ab. 246

185 suriname/ 1082

186 (Suriname or Dutch Guiana).ti,ab. 562

187 venezuela/ 7140

188 Venezuela.ti,ab. 6553

189 Djibouti/ 367

190 (Djibouti or French Somaliland).ti,ab. 380

191 exp egypt/ 20361

192 Egypt.ti,ab. 13812

193 morocco/ 7721

194 Morocco.ti,ab. 5329

195 tunisia/ 7147

196 Tunisia.mp. 7460

197 exp palestine/ 2276

198 (Gaza or West Bank or Palestine).ti,ab. 2151

199 algeria/ 5219

200 Algeria.ti,ab. 3986

201 iran/ 55052

202 (Iran or Persia).ti,ab. 33920

203 iraq/ 5371

204 (Iraq or Mesopotamia).ti,ab. 4418

205 jordan/ 3561

206 Jordan.ti,ab. 3815

207 lebanon/ 3052

208 (Lebanon or Lebanese Republic).ti,ab. 2476

209 libya/ 1500

210 Libya.ti,ab. 1028

211 syria/ 1813

212 (Syria or Syrian Arab Republic).ti,ab. 1481

213 exp yemen/ 1825

214 Yemen.ti,ab. 1680

215 afghanistan/ 2224

216 Afghanistan.ti,ab. 2435

217 nepal/ 7842

218 Nepal.ti,ab. 7127

219 exp bangladesh/ 13341

220 Bangladesh.ti,ab. 12394

221 bhutan/ 598

222 Bhutan.ti,ab. 572

223 exp india/ 134898

224 India.ti,ab. 89409

225 exp pakistan/ 19186

226 Pakistan.ti,ab. 15057

227 maldives/ 245

228 Maldives.ti,ab. 219

229 sri lanka/ 7225

230 (Sri Lanka or Ceylon).ti,ab. 6885

231 angola/ 1562

232 Angola.ti,ab. 1616

233 cameroon/ 7898

234 (Cameroon or Kamerun or Cameroun).ti,ab. 7021

235 cape verde/ 337

236 (Cape Verde or Cabo Verde).ti,ab. 368

237 exp comoros/ 325

238 (Comoros or Glorioso Islands or Mayotte).ti,ab. 477

239 congo/ 2288

240 (Congo not ((Democratic Republic adj3 Congo) or congo red or crimean-congo)).ti,ab. 5151

241 cote d'ivoire/ 4880

242 (Cote d'Ivoire or Cote dIvoire or Ivory Coast).ti,ab. 4833

243 swaziland/ 876

244 (eSwatini or Swaziland).ti,ab. 919

245 ghana/ 11475

246 (Ghana or Gold Coast).ti,ab. 10846

247 kenya/ 19604

248 (Kenya or East Africa Protectorate).ti,ab. 17779

249 lesotho/ 683

250 (Lesotho or Basutoland).ti,ab. 672

251 mauritania/ 579

252 Mauritania.ti,ab. 633

253 nigeria/ 37250

254 Nigeria.ti,ab. 31930

255 exp "sao tome and principe"/ 238

256 (Sao Tome adj2 Principe).ti,ab. 131

257 senegal/ 6820

258 Senegal.ti,ab. 5862

259 sudan/ 7157

260 (Sudan not South Sudan).ti,ab. 7697

261 zambia/ 5721

262 (Zambia or Northern Rhodesia).ti,ab. 5122

263 zimbabwe/ 6962

264 (Zimbabwe or Southern Rhodesia).ti,ab. 5099

265 botswana/ 2156

266 (Botswana or Bechuanaland or Kalahari).ti,ab. 2148

267 equatorial guinea/ 444

268 (Equatorial Guinea or Spanish Guinea).ti,ab. 455

269 gabon/ 1825

270 (Gabon or Gabonese Republic).ti,ab. 1725

271 mauritius/ 1012

272 (Mauritius or Agalega Islands).ti,ab. 991

273 namibia/ 1201

274 (Namibia or German South West Africa).ti,ab. 977

275 exp south africa/ 35848

276 (South Africa or Cape Colony or British Bechuanaland or Boer Republics or Zululand or Transvaal or Natalia Republic or Orange Free State).ti,ab. 26196

277 benin/ 2926

278 (Benin or Dahomey).ti,ab. 3789

279 burkina faso/ 5248

280 (Burkina Faso or Burkina Fasso or Upper Volta).ti,ab. 5187

281 burundi/ 773

282 (Burundi or Ruanda-Urundi).ti,ab. 1045

283 central african republic/ 1151

284 (Central African Republic or Ubangi-Shari).ti,ab. 1168

285 chad/ 1320

286 Chad.ti,ab. 1354

287 congo democratic republic/ 7607

288 (((Democratic Republic or DR) adj2 Congo) or Congo-Kinshasa or Belgian Congo or Zaire or Congo Free State).ti,ab. 6354

289 eritrea/ 741

290 Eritrea.ti,ab. 700

291 ethiopia/ 16027

292 (Ethiopia or Abyssinia).ti,ab. 15104

293 gambia/ 3070

294 Gambia.ti,ab. 2664

295 guinea/ 1323

296 (Guinea not (New Guinea or Guinea Pig* or Guinea Fowl or Guinea-Bissau or Portuguese Guinea or Equatorial Guinea)).ti,ab. 3069

297 guinea-bissau/ 1066

298 (Guinea-Bissau or Portuguese Guinea).ti,ab. 1048

299 liberia/ 1757

300 madagascar/ 4080

301 (Madagascar or Malagasy Republic).ti,ab. 4062

302 malawi/ 7037

303 (Malawi or Nyasaland).ti,ab. 6518

304 mali/ 3427

305 Mali.ti,ab. 3332

306 exp mozambique/ 3446

307 (Mozambique or Mocambique or Portuguese East Africa).ti,ab. 3428

308 niger/ 1419

309 (Niger not (Aspergillus or Peptococcus or Schizothorax or Cruciferae or Gobius or Lasius or Agelastes or Melanosuchus or radish or Parastromateus or Orius or Apergillus or Parastromateus or Stomoxys)).ti,ab. 3729

310 rwanda/ 2650

311 (Rwanda or Ruanda).ti,ab. 2619

312 sierra leone/ 2417

313 (Sierra Leone or Salone).ti,ab. 2429

314 somalia/ 1891

315 (Somalia or Somaliland).ti,ab. 1656

316 south sudan/ 443

317 South Sudan.ti,ab. 555

318 exp tanzania/ 14613

319 (Tanzania or Tanganyika or Zanzibar).ti,ab. 13890

320 togo/ 1808

321 (Togo or Togolese Republic or Togoland).ti,ab. 1699

322 uganda/ 14820

323 Uganda.ti,ab. 13995

324 "africa south of sahara"/ or central africa/ or east africa/ or southern africa/ or west africa/ or tropical africa/ 230818

325 ("Africa South of the Sahara" or sub-Saharan Africa or subSaharan Africa).ti,ab. 16370

326 Central Africa.ti,ab. 2573

327 Eastern Africa.ti,ab. 642

328 Southern Africa.ti,ab. 2956

329 Western Africa.ti,ab. 510

330 or/32-329 [ALL LOW AND MIDDLE INCOME COUNTRIES] 1205212

331 29 and 30 and 31 and 330 41212

332 limit 331 to (english language and full text and yr="2000 -Current") 2123

<https://ovidsp.ovid.com/ovidweb.cgi?T=JS&NEWS=N&PAGE=main&SHAREDSEARCHID=45UyD0P8GnJHjTJhuzSNERsefhzsePMWgzdQyZpXDvYvwNfRHdXjqfuW8COf43ceq>

Social Policy and Practice <202104>

1 (corrupt* or indulg* or absen* or brib* or kickback* or collu* or theft or steal* or illegal or crime or stole* or diver* or "pseudo-trial" or "pseudo trial" or fals* or fake or fraud* or counterfeit or nepotism or "informal pay" or "unnecessary refer*" or dishonest* or "informal practi*" or "illegal practi*" or "illicit practi*" or "speed up money" or "grease money" or "rent seek*" or embezzle* or extort* or skim* or gratuit* or "taint* loan*" or "bid rig*" or misappropriat* or conceal* or "price fix*" or "forc* labo?r" or inept* or "function abus*" or impunit* or squander* or larceny or cronyism or chisel* or patronage or favo?ritism or (abuse adj3 discretion) or (abuse adj3 power) or (crime adj3 "white-collar") or (crime adj3 "white collar") or launder* or neglig* or coerc* or deceiv* or deception or ghost* or blackmail* or clientelism or "private gain*" or (conflict* adj3 interest*)).mp. [mp=abstract, title, publication type, heading word, accession number] 28390

2 (method* or measur* or detect* or surveill* or metric* or indicat* or survey* or interview* or scal* or audit* or monitor* or inquir* or "focus group*" or "discussion group*" or questionnaire* or report* or experiment* or trial* or test* or observ* or assess* or scor* or investig* or qualitative or quanti*).mp. [mp=abstract, title, publication type, heading word, accession number] 216300

3 (((health* or "health-care" or frontline) adj3 (sector* or setting or cent* or facilit* or service* or system* or worker* or staff or provider* or unit* or personnel or deliver*)) or "healthcare" or health or hospital* or manag* or administrat* or "service provider*" or provider* or doctor* or physician* or nurs* or pharmac* or licen* or medic*).mp. [mp=abstract, title, publication type, heading word, accession number] 166158

4 developing country/ or low income country/ or middle income country/ 0

5 ((developing or less* developed or under developed or underdeveloped or middle income or low* income) adj (economy or economies)).ti,ab. 13

6 ((developing or less* developed or under developed or underdeveloped or middle income or low* income or underserved or under served or deprived or poor*) adj (countr* or nation? or population? or world)).ti,ab. 1094

7 (low* adj (gdp or gnp or gross domestic or gross national)).ti,ab. 4

8 (low adj3 middle adj3 countr*).ti,ab. 92

9 (lmic or lmics or third world or lami countr*).ti,ab. 105

10 transitional countr*.ti,ab. 5

11 global south.ti,ab. 32

12 "Africa south of the Sahara"/ 0

13 ("Africa South of the Sahara" or sub-Saharan Africa or subSaharan Africa).ti,ab. 117

14 Central Africa.ti,ab. 3

15 Eastern Africa.ti,ab. 1

16 Southern Africa.ti,ab. 23

17 Western Africa.ti,ab. 4

18 North Korea/ 0

19 (North Korea or (Democratic People* Republic adj2 Korea)).ti,ab. 2

20 Haiti/ 0

21 (Haiti or Hayti).ti,ab. 19

22 Afghanistan/ 0

23 Afghanistan.ti,ab. 67

24 Nepal/ 0

25 Nepal.ti,ab. 43

26 Syrian Arab Republic/ 0

27 (Syria or Syrian Arab Republic).ti,ab. 15

28 Yemen/ 0

29 Yemen.ti,ab. 9

30 Tajikistan/ 0

31 Tajikistan.ti,ab. 8

32 Benin/ 0

33 (Benin or Dahomey).ti,ab. 0

34 Burkina Faso/ 0

35 (Burkina Faso or Burkina Fasso or Upper Volta).ti,ab. 10

36 Burundi/ 0

37 (Burundi or Ruanda-Urundi).ti,ab. 3

38 Central African Republic/ 0

39 (Central African Republic or Ubangi-Shari).ti,ab. 1

40 Chad/ 0

41 Chad.ti,ab. 1

42 Democratic Republic Congo/ 0

43 (((Democratic Republic or DR) adj2 Congo) or Congo-Kinshasa or Belgian Congo or Zaire or Congo Free State).ti,ab. 10

44 Eritrea/ 0

45 Eritrea.ti,ab. 19

46 Ethiopia/ 0

47 (Ethiopia or Abyssinia).ti,ab. 50

48 Gambia/ 0

49 Gambia.ti,ab. 3

50 Guinea/ 0

51 (Guinea not (New Guinea or Guinea Pig* or Guinea Fowl or Guinea-Bissau or Portuguese Guinea or Equatorial Guinea)).ti,ab. 3

52 Guinea-Bissau/ 0

53 (Guinea-Bissau or Portuguese Guinea).ti,ab. 2

54 Liberia/ 0

55 Liberia.ti,ab. 6

56 Madagascar/ 0

57 (Madagascar or Malagasy Republic).ti,ab. 5

58 Malawi/ 0

59 (Malawi or Nyasaland).ti,ab. 34

60 Mali/ 0

61 Mali.ti,ab. 12

62 Mozambique/ 0

63 (Mozambique or Mocambique or Portuguese East Africa).ti,ab. 20

64 Niger/ 0

65 (Niger not (Aspergillus or Peptococcus or Schizothorax or Cruciferae or Gobius or Lasius or Agelastes or Melanosuchus or radish or Parastromateus or Orius or Apergillus or Parastromateus or Stomoxys)).ti,ab. 3

66 Rwanda/ 0

67 (Rwanda or Ruanda).ti,ab. 25

68 Sierra Leone/ 0

69 (Sierra Leone or Salone).ti,ab. 17

70 Somalia/ 0

71 (Somalia or Somaliland).ti,ab. 32

72 south sudan/ 0

73 South Sudan.ti,ab. 2

74 Tanzania/ 0

75 (Tanzania or Tanganyika or Zanzibar).ti,ab. 54

76 Togo/ 0

77 (Togo or Togolese Republic or Togoland).ti,ab. 6

78 Uganda/ 0

79 Uganda.ti,ab. 85

80 Cambodia/ 0

81 Cambodia.ti,ab. 29

82 exp Indonesia/ 0

83 (Indonesia or Dutch East Indies).ti,ab. 73

84 kiribati/ 0

85 (Kiribati or Gilbert Islands or Phoenix Islands or Line Islands).ti,ab. 0

86 Laos/ 0

87 (Laos or (Lao adj1 Democratic Republic)).ti,ab. 6

88 exp "Federated States of Micronesia"/ 0

89 Micronesia.ti,ab. 0

90 Mongolia/ 0

91 Mongolia.ti,ab. 10

92 Myanmar/ 0

93 (Myanmar or Burma).ti,ab. 13

94 Papua New Guinea/ 0

95 (Papua New Guinea or German New Guinea or British New Guinea or Territory of Papua).ti,ab. 7

96 Philippines/ 0

97 (Philippines or Philippine Islands).ti,ab. 80

98 solomon islands/ 0

99 Solomon Islands.ti,ab. 1

100 Timor-Leste/ 0

101 (Timor-Leste or East Timor or Portuguese Timor).ti,ab. 1

102 Vanuatu/ 0

103 (Vanuatu or New Hebrides).ti,ab. 2

104 Viet Nam/ 0

105 (Viet Nam or Vietnam or French Indochina).ti,ab. 93

106 Kyrgyzstan/ 0

107 (Kyrgyzstan or Kyrgyz Republic or Kirghizia or Kirghiz).ti,ab. 11

108 Moldova/ 0

109 Moldova.ti,ab. 17

110 exp Ukraine/ 0

111 Ukraine.ti,ab. 59

112 exp Uzbekistan/ 0

113 Uzbekistan.ti,ab. 8

114 Bolivia/ 0

115 Bolivia.ti,ab. 21

116 El Salvador/ 0

117 El Salvador.ti,ab. 15

118 Honduras/ 0

119 Honduras.ti,ab. 12

120 Nicaragua/ 0

121 Nicaragua.ti,ab. 18

122 Djibouti/ 0

123 (Djibouti or French Somaliland).ti,ab. 0

124 Egypt/ 0

125 Egypt.ti,ab. 47

126 Morocco/ 0

127 Morocco.ti,ab. 13

128 Tunisia/ 0

129 Tunisia.mp. 8

130 palestine/ 0

131 (Gaza or West Bank or Palestine).ti,ab. 56

132 Bangladesh/ 0

133 Bangladesh.ti,ab. 129

134 Bhutan/ 0

135 Bhutan.ti,ab. 4

136 exp India/ 0

137 India.ti,ab. 785

138 exp Pakistan/ 0

139 Pakistan.ti,ab. 115

140 Angola/ 0

141 Angola.ti,ab. 7

142 Cameroon/ 0

143 (Cameroon or Kamerun or Cameroun).ti,ab. 5

144 Cape Verde/ 0

145 (Cape Verde or Cabo Verde).ti,ab. 0

146 Comoros/ 0

147 (Comoros or Glorioso Islands or Mayotte).ti,ab. 0

148 Congo/ 0

149 (Congo not ((Democratic Republic adj3 Congo) or congo red or crimean-congo)).ti,ab. 6

150 Cote d'Ivoire/ 0

151 (Cote d'Ivoire or Cote dIvoire or Ivory Coast).ti,ab. 4

152 eswatini/ 0

153 (eSwatini or Swaziland).ti,ab. 7

154 Ghana/ 0

155 (Ghana or Gold Coast).ti,ab. 119

156 Kenya/ 0

157 (Kenya or East Africa Protectorate).ti,ab. 87

158 Lesotho/ 0

159 (Lesotho or Basutoland).ti,ab. 19

160 Mauritania/ 0

161 Mauritania.ti,ab. 1

162 Nigeria/ 0

163 Nigeria.ti,ab. 135

164 "sao tome and principe"/ 0

165 (Sao Tome adj2 Principe).ti,ab. 0

166 Senegal/ 0

167 Senegal.ti,ab. 15

168 Sudan/ 0

169 (Sudan not South Sudan).ti,ab. 24

170 Zambia/ 0

171 (Zambia or Northern Rhodesia).ti,ab. 29

172 Zimbabwe/ 0

173 (Zimbabwe or Southern Rhodesia).ti,ab. 82

174 American Samoa/ 0

175 American Samoa.ti,ab. 1

176 china/ or guangxi/ or inner mongolia/ or macao/ or ningxia/ or tibet/ or xinjiang/ 0

177 China.ti,ab. 882

178 Fiji/ 0

179 Fiji.ti,ab. 21

180 exp Malaysia/ 0

181 (Malaysia or Malayan Union or Malaya).ti,ab. 102

182 marshall islands/ 0

183 Marshall Islands.ti,ab. 4

184 nauru/ 0

185 Nauru.ti,ab. 0

186 Samoa/ 0

187 ((Samoa not American Samoa) or Western Samoa or Navigator Islands or Samoan Islands).ti,ab. 5

188 Thailand/ 0

189 (Thailand or Siam).ti,ab. 135

190 Tonga/ 0

191 Tonga.ti,ab. 4

192 tuvalu/ 0

193 (Tuvalu or Ellice Islands).ti,ab. 0

194 Albania/ 0

195 Albania.ti,ab. 42

196 Armenia/ 0

197 Armenia.ti,ab. 21

198 exp Azerbaijan/ 0

199 Azerbaijan.ti,ab. 12

200 Belarus/ 0

201 (Belarus or Byelarus or Byelorussia or Belorussia).ti,ab. 11

202 exp "Bosnia and Herzegovina"/ 0

203 (Bosnia or Herzegovina).ti,ab. 41

204 Bulgaria/ 0

205 Bulgaria.ti,ab. 119

206 exp "Georgia (republic)"/ 0

207 Georgia.ti,ab. not "georgia (u.s.)"/ 0

208 Kazakhstan/ 0

209 (Kazakhstan or Kazakh).ti,ab. 15

210 Kosovo/ 0

211 Kosovo.ti,ab. 41

212 "Montenegro (republic)"/ 0

213 Montenegro.ti,ab. 7

214 "republic of north macedonia"/ 0

215 North Macedonia.ti,ab. 1

216 Romania/ 0

217 Romania.ti,ab. 212

218 exp Russian Federation/ 0

219 ussr/ 0

220 (Russia or Russian Federation or USSR or Union of Soviet Socialist Republics or Soviet Union).ti,ab. 363

221 exp Serbia/ 0

222 Serbia.ti,ab. 32

223 "Turkey (republic)"/ 0

224 (Turkey.ti,ab. not "Turkey (bird)"/) or (Anatolia or Asia Minor).ti,ab. 0

225 Turkmenistan/ 0

226 Turkmenistan.ti,ab. 4

227 Argentina/ 0

228 (Argentina or Argentine Republic).ti,ab. 53

229 Belize/ 0

230 (Belize or British Honduras).ti,ab. 4

231 exp Brazil/ 0

232 Brazil.ti,ab. 188

233 Colombia/ 0

234 Colombia.ti,ab. 40

235 Costa Rica/ 0

236 Costa Rica.ti,ab. 17

237 Cuba/ 0

238 Cuba.ti,ab. 29

239 Dominica/ 0

240 Dominica.ti,ab. 1

241 Dominican Republic/ 0

242 Dominican Republic.ti,ab. 19

243 Ecuador/ 0

244 Ecuador.ti,ab. 11

245 Grenada/ 0

246 Grenada.ti,ab. 7

247 Guatemala/ 0

248 Guatemala.ti,ab. 29

249 Guyana/ 0

250 (Guyana or British Guiana).ti,ab. 6

251 Jamaica/ 0

252 Jamaica.ti,ab. 45

253 exp Mexico/ 0

254 (Mexico or United Mexican States).ti,ab. 235

255 Paraguay/ 0

256 Paraguay.mp. 1

257 Peru/ 0

258 Peru.ti,ab. 36

259 Saint Lucia/ 0

260 (St Lucia or Saint Lucia or Iyonala or Hewanorra).ti,ab. 3

261 "Saint Vincent and the Grenadines"/ 0

262 (Saint Vincent or St Vincent or Grenadines).ti,ab. 11

263 Suriname/ 0

264 (Suriname or Dutch Guiana).ti,ab. 2

265 Venezuela/ 0

266 Venezuela.ti,ab. 10

267 Algeria/ 0

268 Algeria.ti,ab. 13

269 Iran/ 0

270 (Iran or Persia).ti,ab. 93

271 exp Iraq/ 0

272 (Iraq or Mesopotamia).ti,ab. 90

273 Jordan/ 0

274 Jordan.ti,ab. 41

275 Lebanon/ 0

276 (Lebanon or Lebanese Republic).ti,ab. 46

277 Libyan Arab Jamahiriya/ 0

278 libya.ti,ab. 5

279 maldives/ 0

280 Maldives.ti,ab. 1

281 Sri Lanka/ 0

282 (Sri Lanka or Ceylon).ti,ab. 64

283 Botswana/ 0

284 (Botswana or Bechuanaland or Kalahari).ti,ab. 50

285 Equatorial Guinea/ 0

286 (Equatorial Guinea or Spanish Guinea).ti,ab. 0

287 Gabon/ 0

288 (Gabon or Gabonese Republic).ti,ab. 0

289 Mauritius/ 0

290 (Mauritius or Agalega Islands).ti,ab. 4

291 Namibia/ 0

292 (Namibia or German South West Africa).ti,ab. 11

293 South Africa/ 0

294 (South Africa or Cape Colony or British Bechuanaland or Boer Republics or Zululand or Transvaal or Natalia Republic or Orange Free State).ti,ab. 777

295 or/4-294 [ALL LOW AND MIDDLE-INCOME COUNTRIES] 6314

296 1 and 2 and 3 and 295 172

<https://ovidsp.ovid.com/ovidweb.cgi?T=JS&NEWS=N&PAGE=main&SHAREDSEARCHID=1mKSgfhL3on7fTZqxmlbviI0m2p8qhxV6ONtK5fcCoG3I2Vq6GX8beS6MprEHijE2>

Web of Science July 2021

- 1. TS=((corrupt* or indulg* or absen* or brib* or kickback* or collu* or theft or steal* or illegal or crime or stole* or diver* or "pseudo-trial" or "pseudo trial" or fals* or fake or fraud* or counterfeit or nepotism or "informal pay" or "unnecessary refer*" or dishonest* or "informal practi*" or "illegal practi*" or "illicit practi*" or "speed up money" or "grease money" or "rent seek*" or embezzle* or extort* or skim* or gratuit* or "taint* loan*" or "bid rig*" or misappropriat* or conceal* or "price fix*" or "forc* labo?r" or inept* or "function abus*" or impunit* or squander* or larceny or cronyism or chisel* or patronage or favo?ritism or (abuse adj3 discretion) or (abuse adj3 power) or (crime adj3 "white-collar") or (crime adj3 "white collar") or launder* or neglig* or coerc* or deceiv* or deception or ghost* or blackmail* or clientelism or "private gain*" or (conflict* adj3 interest*)))

3,308,873

- 1. TI=((method* or measur* or detect* or surveill* or metric* or indicat* or survey* or interview* or scal* or audit* or monitor* or inquir* or "focus group*" or "discussion group*" or questionnaire* or report* or experiment* or trial* or test* or observ* or assess* or scor* or investig* or qualitative or quanti*))

9,296,163

- 1. TI=(((health* or "health-care" or frontline) adj3 (sector* or setting or cent* or facilit* or service* or system* or worker* or staff or provider* or unit* or personnel or deliver*)) or "healthcare" or health or hospital* or manag* or administrat* or "service provider*" or provider* or doctor* or physician* or nurs* or pharmac* or licen* or medic*)

3,130,559

- 1. TS=((countr* adj3 (“low-income” or “low income” or “middle-income” or “middle income”)) or (setting* adj3 (“low-income” or “low income” or “middle-income” or “middle income”)) or “low income” or “middle income” or “low-income” or “low income” or “develop* countr*” or LMIC or MIC or LIC or LMICS or MICS or LICS)

322,925

- 1. (((#1) AND #2) AND #3) AND #4

767

1. A((#1) AND #2) AND #3) AND #4 and English (Languages)

761

1. A((#1) AND #2) AND #3) AND #4 and English (Languages) and 2021 or 2020 or 2019 or 2018 or 2017 or 2016 or 2015 or 2014 or 2013 or 2012 or 2011 or 2010 or 2009 or 2008 or 2007 or 2006 or 2005 or 2004 or 2003 or 2002 or 2001 or 2000

739

<https://www.webofscience.com/wos/woscc/summary/a99b772c-7b75-4f88-97fa-e3c53ee6b508-032e881b/relevance/1>

**Appendix C. Methods for Measuring Corruption (UNDP, 2015; Vian, 2008) – a Summary**

| **Method** | **Advantages** | **Disadvantages** |
| --- | --- | --- |
| Surveys | - Can be offered to a range of different actors. - Capture the prevalence, experiences, and perceptions of corrupt practices; can provide descriptive and explanatory accounts. | - Expensive and labour-intensive - Possible limited in validity for capturing corruption given varying definitions of corruption |
| Expert surveys | - Capture the nature of corruption and the quality of systems which it affects - Less resource intensive than traditional surveys | - Difficulty locating and accessing experts; these stakeholders may not be willing to discuss a sensitive topic such as corruption - Dispersion of experts can hinder intra-country comparison - Potentially poor representativeness |
| Monitoring and evaluation systems | - Investigate government practices and international development initiatives can produce policy-relevant data for anticorruption strategies - Identify system limitations that allow corrupt practices to persist | - Quality and standardization of data collection may hinder cross-country comparisons - Corrupt practices may impact the quality of data and render such practices difficult to detect |
| Crowdsourcing | - Bolster citizen knowledge - Gather ideas from beyond experts, such as laypersons - Capture real-time experiences of corruption | - Results depend on the quality of entries - Difficult to achieve anonymity; this may hinder the information sharing for a corruption, a highly sensitive topic |
| Compliance review/tests | - Evaluate transparency and accountability systems and their impact on corruption - Assess how well governments and organizations are complying with their own policies and regulations to prevent corrupt practices | - Costly to conduct - Corruption may impact the reporting of organization and government practices, therefore limiting the validity of results |
| Indicator/Scorecard-driven case studies | - Reduce the need to code immense amounts of data post-collection | - Accuracy and validity of results depend on subjective interpretation |
| Control systems review | - Compare actual systems with best practices - Yield deep analysis of specific governmental/state departments or units and their engagement in or refrain from corruption | - Assumes that systems are stable, thus unsuitable for systems in transition - Usefulness of results dependent upon effective administrative systems and quality documentation |
| Qualitative data collection | - Elicit data on attitudes, norms, beliefs, and pressures surrounding corrupt practices - Define terms, clarifying the ‘how’ of corruption, and inform production of perceptions surveys - Follow up with participants | - Data influenceable by social desirability and recall biases, thus can underestimate the extent of corruption and its impacts - Resources needed to train research personnel, to produce full cross-cultural meaning of corruption and results |
| Household and public expenditures surveys | - Garner detail on household spending on health, stratified by various factors, to indicate the prevalence of corrupt practices - Compare data with targets to provide measure of accountability and the extent of corrupt practices | - Data may not have been collected in a way that differentiates between formal and informal payments - Public expenditure surveys rely on public records, and these may be inconsistent |

**Appendix D. Study Extraction Table (non-abbreviated)**

| **Study Title** | **Study (by author, date)** | **Country** | **Sampling** | **Methods Used** | **Purpose and Setting** | **Was Measuring HSC the Main Study Focus?** | **Aspect(s) of Corruption Elicited (type of results)** | **Strengths and Limitations of Measurement Method (as reported)** |
| --- | --- | --- | --- | --- | --- | --- | --- | --- |
| 1.The impact of delays on maternal and neonatal outcomes in Ugandan public health facilities: the role of absenteeism | Ackers et al., 2016 | Uganda | Inadequately described | Quantitative: audit of health performance data  Qualitative: interviews; focus groups | Assess the role of healthcare worker absenteeism on the quality of maternal and neonatal care in public healthcare facilities. | N | - Frequency - Drivers - Impacts | None stated. |
| 2.Private money-making indulgence and inefficiency of primary healthcare in Nigeria: a qualitative study of health workers’ absenteeism | Agwu et al., 2020 | Nigeria | Purposive for study sites; convenience for interviewees | Qualitative: in-depth key informant interviews | Explore the prevalence and extent of absenteeism and indulgence in the primary healthcare setting. | Y | - Prevalence - Drivers - Perceptions - Potential remedies | It was difficult to discern whether survival absenteeism constitutes corruption, given that the need to miss work to survive economic difficulty makes the individual vulnerable and absenteeism becomes a method of economic protection. |
| 3.Incentivising doctor attendance in rural Bangladesh: a latent class analysis of a discrete choice experiment. | Angell et al., 2021 | Bangladesh | Inadequately described | Quantitative: discrete choice experiment | Examine doctors’ preferences regarding their jobs, to inform policy interventions for preventing absenteeism. | N | - Drivers - Perceptions - Susceptible actors | Discrete choice experiment results reflect respondents' preferences towards hypothetical situations, rather than real-world actions. Results could be biased if the stated choices were different from how respondents would act in reality, and this could be especially relevant for a sensitive topic like absenteeism. The impact of this possible bias was minimized through measured including the option for respondents to opt-out, and a rigorous and evidence-informed attribute generation process, as well as attributes being piloted. |
| 4.Governance in managing public health resources in Brazilian municipalities | Avelino et al., 2014 | Brazil | Probability | Quantitative: review of health programme audit reports | Investigate fraud in procurement, diversion of public funds for private gain, and over-invoicing for health-related goods and services, in municipalities. | N | - Prevalence - Extent (financial) | Auditor reports overcome the subjectivity of other corruption indicators. |
| 5.Understanding informal payments for health care: the example of Bulgaria | Balabanova & McKee, 2002 | Bulgaria | Random | Quantitative: surveys  Qualitative: focus groups | Investigate the practice of informal payments for healthcare, as well as perceptions towards them. | Y | - Prevalence - Types - Correlates - Drivers | Given the sensitive nature of the topic, as well as the possibility for recall bias, some underreporting may have occurred. Additionally, some aspects of informal payments were elucidated after the research started; earlier, this knowledge could have improved the survey and its distinction between different types of payments. |
| 6.Diffusion of counterfeit drugs in developing countries and stability of galenics stored for months under different conditions of temperature and relative humidity | Baratta et al., 2012 | Angola, Brazil, Cameroun, Central African Republic, Chad, Congo, Ethiopia, Guinea Bissau, Guinea Conakry, India, Kenya, Madagascar, Malawi, Rwanda, and Uganda. | Inadequately described | Quantitative: sampling of medicines for quality and authenticity | Assess the prevalence of counterfeit medicines in pharmacies and unofficial street pharmacies. | Y | - Prevalence | The scientific (chemical and physical) analysis in this paper was deemed a suitable method for assessing the quality of medicines; and moreover, it was considered ideal for developing countries thanks to its low costs and simplicity. |
| 7.Corruption in the health care sector: A barrier to access of orthopaedic care and medical devices in Uganda | Bouchard et al., 2012 | Uganda | Snowball | Qualitative: open-ended semi-structured interviews | Analyze the impact of corruption on access to orthopaedic care and medical devices. | Y | - Prevalence - Locations - Types - Drivers | Open-ended interviews and case studies were considered less prone to bias than other qualitative methods because preconceived theories and beliefs are difficult to impose on the data. However, snowball sampling may had introduced bias since people with similar views may be more likely to know each other. Restricting this bias, however, was the fact that more than half of the orthopaedic surgeons from the country were interviewed. |
| 8.Ghost Doctors: Absenteeism in Rural Bangladeshi Health Facilities | Chaudhury & Hammer, 2004 | Bangladesh | Random | Quantitative: unannounced visits to healthcare facilities | Measure absenteeism amongst medical professionals in rural healthcare facilities. | Y | - Prevalence - Locations - Correlates | Results were mostly descriptive; causal associations could not be confirmed. Data from surprise visits were collected infrequently and therefore limited in their ability to discern reasons for attendance rates and trends. Further, collecting more comprehensive data from absent workers would have been more expensive. This study did provide important information about who are important actors in the issue, to inform future work. |
| 9.The impact of hospital management reforms on absenteeism in Costa Rica | Garcia-Prado & Chawla, 2006 | Costa Rica | ‘Mainly’ random | Quantitative: review of healthcare worker absence records | Assess healthcare worker absenteeism rates among hospitals, given recent hospital management reforms. | Y | - Prevalence - Impacts of hospital management interventions | Given that the data were a collection of all unscheduled absences, it was impossible to distinguish between voluntary and involuntary absences. Further, the authors were lacking data regarding the length of absences, differences between males and females, and differences between different types of healthcare workers. By contrast, the data was sourced from the Social Security Institute and included a large number of variables and indicators from across the country’s health sector. |
| 10.Over-the-counter antibiotic sale in community and online pharmacies, China | Gong et al., 2020 | China | Multi-stage random | Quantitative: assessment of antibiotic sales practices via simulated clients | Assess the practice of illegally selling antibiotics among online and in-person (community) pharmacies. | Y | - Prevalence - Correlates | The study evaluated requests for antibiotics only, which constitutes a small portion of total pharmacy sales and may underestimate the prevalence of illegal antibiotic dispensing. |
| 11.Identification of Substandard and Falsified Medicines: Influence of Different Tolerance Limits and Use of Authenticity Inquiries | Hauk et al., 2021 | Cameroon, Democratic Republic of the Congo, and Malawi | Inadequately described | Quantitative: sampling of medicines for quality and authenticity | Investigate the prevalence of substandard and falsified medicines. | Y | - Prevalence | Authenticity inquiry was noted as constituting high workload for researchers, manufacturers, and distributors, despite being a useful strategy/tool. Manufacturers may be inclined to be deceitful and low response rates can affect the authenticity inquiry. The authors noted that studies of this sort should clearly distinguish between substandard and falsified medications, especially when categorizing for scientific purposes. |
| 12.Do they work? Regulating for-profit providers in Zimbabwe | Hongoro & Kumaranayake, 2000 | Zimbabwe | Inadequately described | Qualitative: structured interviews using open-ended questions | Explore health-sector stakeholders’ knowledge and concerns regarding healthcare laws and regulations. | N | - Prevalence - Types - Drivers | None stated. |
| 13.Good governance and corruption in the health sector: lessons from the Karnataka experience | Huss et al., 2011 | India | Inadequately described | Qualitative: semi-structured interviews | Analyze governance practices and corrupt behaviours in the health sector. | N | - Prevalence - Drivers - Types - Changes over time | The authors noted that, on several occasions, it was impossible to distinguish between mismanagement, misjudgment, and corruption. |
| 14.Targeting anticorruption interventions at the front line: developmental governance in health systems. | Hutchinson et al., 2020 | Bangladesh | Inadequately described | Qualitative: in-depth interviews with doctors in rural Bangladesh | Understand how doctor absenteeism is shaped by policy and health systems conditions, as well as political and social networks. | N | - Prevalence - Perceptions - Drivers | None stated. |
| 15.Informal payments and health worker effort: a quantitative study from Tanzania | Ida Lindkvist, 2012 | Tanzania | Random | Qualitative: interviews | Examine informal payment practices in healthcare facilities. | N | - Prevalence | Patients may have been reluctant to share information about informal payments and such practices. Further, patients may have been unaware of these practices. The authors noted the possibility of classifying health workers as accepting informal payments, if enough patients say they do. However, they noted that this could be difficult to implement if some workers see a limited number of patients., given that researchers would be approaching only patients who are not willing or unable to describe this practice. |
| 16.An investigation into the Quality of Medicines in Yangon, Myanmar | Islam et al., 2018 | Myanmar | Inadequately described | Quantitative: sampling of medicines for quality and authenticity | Evaluate the existence of substandard and falsified medicines across pharmacies, hospitals, and wholesalers. | Y | - Prevalence - Locations | None stated. |
| 17.Improving Fraud Abuse and Detection in General Physician Claims: A Data Mining Study | Joudaki et al., 2016 | Iran | Inadequately described | Quantitative: review of insurance data  Qualitative: expert interviews | Investigate occurrences of fraud, abuse, and collusion from general physician claims. | N | - Prevalence - Indicators | Data may require information system restructuring; moreover, some essential data from fraud and abuse may have been poorly recorded. In settings with multiple insurers, the segmentation of insurance markets could hinder the ability to accurately detect fraud and abuse. Using physicians as the unit of analysis, instead of claims, made data mining more manageable and potentially more suitable for practice in low-resource settings. |
| 18.A cross-sectional analysis of falsified, counterfeit and substandard medicines in a low-middle income country | Khurelbat et al., 2020 | Mongolia | Mix of random and convenience | Quantitative: sampling of medicines for quality and authenticity | Evaluate the presence of substandard and falsified medicines across pharmacy wholesalers. | Y | - Prevalence - Locations | Samples with suspicious packaging and labelling were sent to manufacturers to confirm authenticity, yet falsified products could have been produced in the same facility as licensed equivalents. |
| 19.The performance of health workers in Ethiopia: Results from qualitative research | Lindelow & Serneels, 2006 | Ethiopia | Inadequately described | Qualitative: semi-structured focus groups | Assess the performance of healthcare workers, particularly factors which impede performance and quality of services. | N | - Prevalence - Types - Drivers | It was noted that focus groups can elicit views not easily obtained through individual interviews. The authors noted that focus groups can be useful for exploring sensitive issues like corruption. It was claimed by the authors that focus groups, however, are not objective or representative and findings are influenced by the applied methodology. |
| 20.Surveillance for falsified and substandard medicines in Africa and Aisia by local organization using the low-cost GPHF Minilab | Peterson et al., 2017 | Cameroon, Democratic Republic of the Congo, India, Ghana, Kenya, Nigeria, and Uganda | Convenience | Quantitative: sampling of medicines for quality and authenticity | Evaluate the prevalence of falsified and substandard medicines. | Y | - Prevalence | None stated. |
| 21.Equivalence in Active Pharmaceutical Ingredient of Generic Antihypertensive Medicines Available in Nigeria (EQUIMEDS): A Case for Further Surveillance. | Redfern et al., 2019 | Nigeria | Semi-random | Quantitative: sampling of medicines (via mystery shoppers) for quality  Qualitative: retailer survey | Measure the quality of antihypertensive medicines in retail markets, as well as to elicit retailers’ perspectives on the issue and its correlates. | Y | - Prevalence - Locations - Correlates | Sampling was limited to three states for practical reasons and because of the extent of hypertension in the country. The literature is lacking on information regarding whether substandard medicines only arise from poor manufacturing or due to improper storage or expiration. |
| 22.Exploring corruption in the South African health sector | Rispel et al., 2016 | South Africa | Purposive | Quantitative: review of audit reports  Qualitative: semi-structured key informant interviews; content analysis of print media. | Investigate HSC broadly. | Y | - Prevalence - Types - Locations - Drivers | Audit data was from the public sector only, as such data did not exist for the private sector. The use of irregular expenditures as a proxy for corruption could have produced an overestimate in corrupt practices, since some of these expenditures could have been made in non-corrupt ways. Key informants may have interpreted the term 'corruption' differently and their interview responses reflected their views at a single point in time. The content reflected in print media can be arbitrary. Despite these limitations, all three triangulated methods yielded consensus. |
| 23."Our fear is finished," but nothing changes: efforts of marginalized women to foment state accountability for maternal health care in a context of low state capacity | Schaaf & Dasgupta, 2019 | India | Purposive | Qualitative: in-depth structured interviews; participant observation.  Quantitative: document review | Assess accountability and the quality of maternal health services, as well as to explore informal financial transactions. | N | - Prevalence - Types | Some of the minor results were deemed tepid, due to disagreement between interviewees. |
| 24.Patterns for informal patient payments in Bulgaria, Hungary and Ukraine: a comparison across countries, years and type of services | Stepurko et al., 2017 | Bulgaria, Hungary, and the Ukraine | Multi-stage random | Quantitative: surveys | Evaluate the practice of and experience with informal payments. | Y | - Prevalence - Types - Changes over time | Limitations included the sensitive nature of the questions on informal payments, the cross-sectional design of the survey, and the fact that the study period only covered 2-3 subsequent years. Furthermore, the magnitude of informal payments could not be discerned since data were only collected on annual payments and last hospital visit. Recall bias could also be an issue. Short study period limits the ability to make strong time trend interpretations. |
| 25.To pay or not to pay? A multicountry study on informal payments for health-care services and consumers' perceptions | Stepurko et al., 2015 | Bulgaria, Hungary, Lithuania, Poland, Romania, and Ukraine | Multi-stage random | Quantitative: surveys | Assess patients’ perceptions of and experiences with informal payments. | Y | - Prevalence - Perceptions | Face to face interviews were used to administer the surveys, due to respondent needs. However, it was claimed that self-administration would have been more suitable given the nature of the study topic. Despite this, a maximum of 5% of respondents refused to answer some questions regarding informal payments, thereby suggesting that the topic was of low sensitive nature. |
| 26.Perceptions of per diems in the health sector: evidence and implications | Vian et al., 2012 | Malawi and Uganda | Snowball | Qualitative: semi-structured interviews | Investigate abuse and perceptions of per diems in the health sector. | N | - Prevalence - Types - Correlates | Informants sometimes discussed per diem practices in cultural terms, for instance 'what people here do.’ It was noted that this does not provide evidence for the prevalence or scope of per diem abuse. This study did not quantify the quantity of per diem spending. |
| 27.Patient pathways to tuberculosis diagnosis and treatment in a fragmented health system: a qualitative study from a south Indian district | Yellappa et al., 2017 | India | Purposive | Qualitative: in-depth interviews | Explore the diagnosis and treatment of tuberculosis and identify factors which impact these. | N | - Prevalence | Health seeking trajectories were explained from patient and private provider perspectives. Qualitative methodology facilitated exploration of perceptions and experiences associated with TB care. Patient narratives are subject to recall bias, though. Patients were contacted by programme staff and may have hesitated to share information that could jeopardize their rapports. |
| 28.A Cross-Sectional Investigation for Verification of Globalization of Falsified Medicines in Cambodia, Indicated by Tablets of Sildenafil Citrate | Yoshida et al., 2019 | Cambodia | Mix of random and convenience | Quantitative: sampling of medicines for quality and authenticity | Determine the existence of falsified medicines in drug outlets and wholesalers. | Y | - Prevalence - Locations | Due to insufficient resources, the authors noted that they could not clarify the composition of excipients in the tablets as it pertained to harmful substances that falsified medicines may contain. |
| 29.“Whistleblowing”: a health issue | Lennane, 1993 | Australia | Purposive | Quantitative: surveys | To examine how organisations respond to whistleblowing; how whistleblowers appraise the responses they received, and how they are treated. | N | - Prevalence - Potential remedy | The author mentioned that they received 35 of the 92 questionnaires sent to persons who had used a whistleblowing service. They reported that the reasons for the over 50% decline were due to fear of victimisation and a reluctance to reopen old wounds. Concerns about dominant collusion between authorities and those whom they blew the whistle against, persisted, especially as the corruption they reported continued unabated. |
| 30.Corruption and Growth | Mauro, 1995 | 70 Countries across Africa, Asia, North & South America, Europe, and Oceania | Purposive | Quantitative: analysis of datasets that emerged from the survey of Business International’s correspondents | Identify the several means through which corruption exercises effects on economic growth, and quantify the magnitude of the effects. | N | - Prevalence - Risk factors - Effects | It was stated that working with a multi-country dataset made it possible to conduct a cross-country analysis of bureaucratic inefficiencies across countries from different continents, including their effects on investments in sectors like education and health. |
| 31.Corruption in Latvia: survey evidence | Anderson., 1998 | Latvia | Mix of random and purposive | Quantitative: surveys of household, public officials, and enterprises | To provide a diagnosis of corruption in Latvia, highlighting the complexity of corruption as well as its causes and consequences. | N | - Types - Prevalence - Causes - Effects | Household surveys only elicit sound information on low-level corruption, while reasonable knowledge on high-level or grand corruption can only be arrived at by interacting with public officials and enterprise staff who interface with government officials. |
| 32.Enhancing accountability and responsiveness in public utilities: exploring the potency of public feedback | Gopakumar and Balakrishnan, 2000 | India | Mix of random and purposive | Mixed method: surveys, interviews, and observations | Determine and illustrate the potency of report cards by citizens on the efficient provision of public services. | N | - Nature - Remedy | Mixed method helped reduce the bias of researchers and revealed errors in quantitative measurements because the survey instruments were informed by the qualitative data. |
| 33.Transparency and accountability in Bolivia: Does voice matter? | Gray-Molina., de Rada., and Yanez, 1999 | Bolivia | Inadequately described | Quantitative: survey | Analysing the institutional factors that influence accountability in the provision of health services and the incentives for service providers and users to participate or not participate in corruption. | Y | - Types - Prevalence - Drivers - Solutions | Demonstrated that surveys that collect contributions of citizens’ voices to accountability in public services can gain the attention of reforms. Additionally, they provide an opportunity to examine the diverse sociodemographic influences and experiences associated with corruption. |
| 34.New frontiers in diagnosing and combatting corruption | Kaufmann., Pradhan., and Ryterman, 1998 | Albania, Georgia, and Latvia | Inadequately described | Mixed method: survey and focus groups | Establishing an anticorruption agenda using empirical surveys, including the challenges in translating such data into practice and policy. | N | - Nature - Prevalence - Solutions | Surveys and interviews are useful tools for eliciting information on corruption among public officials. However, responses will typically yield lower-bound estimates and are not capable of correctly estimating the social and economic costs of corruption. To overcome this, adopting multiple methods, including empirical modelling and varied sources of data, is encouraged. |
| 35.Corruption and the composition of government expenditure | Mauro, 1998 | Over 90 countries across Africa, Asia, North & South America, Europe, and Oceania | Inadequately described | Quantitative: surveys (analysis of datasets) | Explores the relationship between each item on government’s public expenditure list and corruption | N | - Effects | A combination of datasets on expenditure with those on corruption, provides nuanced information on the effects of corruption on the spending of government. It highlights expenditure areas with less or more corruption, because of elite interests, type of dominant expenditure (whether capital or recurrent), and extent to which a sector is prone to bribery. However, it demonstrates poor capacity for practical recommendations. |
| 36.Do budgets really matter? Evidence from public spending on education and health in Uganda | Ablo and Reinikka., 1998 | Uganda | Random | Mixed method: survey, focus groups, and observations | To determine why budget allocations were not yielding commensurate service outputs and outcomes | Y | - Types - Effects | Surveys that worked well in schools rarely yielded substantive results on corruption in the health sector. This led to the deployment of qualitative approaches. Results from surveys catalysed government actions and inspired replication of the surveys after a series of interventions. |
| 37.Informal health markets and formal health financing policy in Uganda: final report | Asiimwe., Mwesigye., McPake., and Streefland., 1997 | Uganda | Mix of random and purposive | Mixed method: surveys, interviews, and observations | How health workers devise socioeconomic survival strategies to sustain themselves amidst payment irregularities and the implications of availability and quality of healthcare to citizens. | Y | - Nature - Drivers - Effects - Solutions | A combination of methods provides a holistic picture of the informal behaviours of public health workers that undermine the public health system, including the inflation of healthcare costs and the diversion of drugs and patients for private sales and to private facilities, respectively. This method also provides an opportunity to evaluate cost of living for the households of health workers in comparison to their salaries. However, the authors noted that their methods worked because the health workers were open about their situations, signaling caution about applying the same methods in countries with repressive regimes. |
| 38.Corruption, public investment, and growth | Tanzi and Davoodi., 1998 | Over 90 countries across Africa, Asia, North & South America, Europe, and Oceania | Inadequately described | Quantitative: surveys (analysis of datasets) | The effects of corruption on public investments and the knock-on effects on operation and maintenance costs like healthcare | N | - Effects | With datasets, researchers can pull together diverse variables against corruption to produce convincing arguments about its macro influences. For instance, this study demonstrates that high public investments in countries notorious for corruption tend to increase corruption and reduced productivity. It suggests that increase in public investments did not improve health. However, with datasets, solutions are rarely described. |
| 39.Informal economic activities of public health workers in Uganda: implications for quality and accessibility of care | McPake., Asiimwe., Mwesigye., Ofumbi., Ortenblad., Streefland., and Turinde., 1999 | Uganda | Mix of random and purposive | Mixed method: surveys, interviews, and observations | The informal economic activities health workers undertake within the system away from regulations and their effects on delivery of quality and affordable healthcare to citizens | Y | - Types - Nature - Prevalence - Drivers - Effects | A combination of methods was considered significantly optimal for this study, as the limitations observed in one method are counteracted by the strengths of another. Additionally, questions for the quantitative survey were derived from insights gained from the qualitative data. For instance, to identify drug leakages using surveys, it was best to consider enquiring about ghost patients, as prescriptions were usually made for every ghost patient, with the prescription value ending up in the pockets of providers. |
| 40.Measuring public hospital costs: empirical evidence from the Dominican Republic | Lewis., La Forgia., and Sulvetta., 1996 | Dominican Republic | Random | Quantitative: surveys | Comparing the actual resource costing of provided health services based on reports by service users to the stated cost of expenditure available to the public | Y | - Nature - Cost of corruption - Solutions | Service users’ targeted surveys on costing can yield comparable data to that produced by health providers. This can be useful for tracking low productivity and resource wastage caused by corruption. For instance, despite spending on physicians accounting for the majority of 84% of the budget allocated to personnel, the survey reported that only 12% of physicians’ time was committed to the hospital. |
